# An evaluation of image-based and statistical techniques for harmonizing brain volume measurements

**DOI:** 10.1162/IMAG.a.73

**Published:** 2025-07-14

**Authors:** Yuan-Chiao Lu, Lianrui Zuo, Yi-Yu Chou, Blake E. Dewey, Samuel Remedios, Russell T. Shinohara, Sonya U. Steele, Govind Nair, Daniel S. Reich, Jerry L. Prince, Dzung L. Pham

**Affiliations:** The Henry M Jackson Foundation for the Advancement of Military Medicine, Inc., Bethesda, MD, United States; Department of Radiology and Bioengineering, Uniformed Services University of the Health Sciences, Bethesda, MD, United States; Department of Radiology and Imaging Sciences, National Institutes of Health, Bethesda, MD, United States; Military Traumatic Brain Injury Initiative (MTBI2), Bethesda, MD, United States; Vanderbilt Institute for Surgery and Engineering, Nashville, TN, United States; Department of Neurology, Johns Hopkins University, Baltimore, MD, United States; Department of Electrical and Computer Engineering, Johns Hopkins University, Baltimore, MD, United States; Department of Biostatistics, Epidemiology and Informatics, University of Pennsylvania, Philadelphia, PA, United States; Translational Neuroradiology Section, National Institute of Neurological Disorders and Stroke, Bethesda, MD, United States

**Keywords:** image harmonization, magnetic resonance imaging, brain volumes, segmentation, ComBat, deep learning

## Abstract

Volumetric analysis of magnetic resonance brain images is often complicated by variations in scanner hardware, software, and acquisition settings. Over the past several years, there has been an increase in the use of retrospective harmonization techniques for addressing these variations. This research evaluates three image harmonization methods—neuroCombat (a statistical batch correction tool), DeepHarmony (a supervised deep learning method based on image-to-image translation), and HACA3 (an unsupervised deep learning image translation approach). The study focuses on their effectiveness in achieving consistent brain volume measurements across differing T1-weighted acquisitions (GRE and MPRAGE) and their ability to detect simulated atrophy changes in the same acquisitions. While all three methods notably enhance the consistency of regional brain volumes compared with unharmonized images, HACA3 demonstrated the lowest measurement variation in terms of absolute volume difference percentage (AVDP) across all brain regions (<3%). It also demonstrated the highest agreement between the coefficient of variation (CV) measurements of GRE and MPRAGE images, evidenced by the smallest mean difference (0.12) and the narrowest 95% confidence intervals ([-1.04, 1.28]), alongside achieving the highest intra-class correlation (ICC) values across all regions (ICC >0.9). In the atrophy simulation experiments, HACA3 consistently achieved the smallest AVDPs across most unchanged brain regions, while DeepHarmony showed significant improvements in several regions, and neuroCombat exhibited higher variability. Additionally, using neuroCombat with training data effectively detected hippocampal atrophy, whereas without training, neuroCombat could not differentiate between images with and without atrophy, highlighting a potential limitation in its ability to detect subtle brain volume changes when training data are unavailable. In most metrics, HACA3 was found to be the most effective for harmonizing MRI data, followed by DeepHarmony, with neuroCombat showing more measurement variability but still offering improvements over unharmonized data.

## 1 Introduction

Automated techniques for quantifying brain volumes from magnetic resonance imaging (MRI) have shown great promise in facilitating clinical research studies by providing insights into patterns of brain aging and disease. However, these approaches are frequently limited by the availability of training datasets, and, as a result, cannot capture the diversity of all potential input images. Such methods perform inaccurately and inconsistently when faced with imaging data with varying acquisition properties that can occur due to scanner changes, software upgrades, and evolving imaging protocols (Pinto et al., 2020). These variations are exacerbated in multi-center research studies, which are increasingly important for enhancing statistical power and population diversity. Methods to reduce variations in the collected data are known as harmonization methods (Dennis et al., 2023). One approach to harmonization is to standardize the MRI acquisition prospectively. However, this approach has shown limitations in its efficacy and is often impractical due to the heterogeneous resources available at different scan sites (Clark et al., 2023). Therefore, alternative methods are needed for the reliable comparison and analysis of heterogeneous MRI data across sites and over time.

In the absence of effective standardization prospectively to address the heterogeneity commonly present in MRI datasets, two primary approaches can be employed to obtain accurate volumetric measurements. The first approach involves using segmentation algorithms that are either robust to acquisition variations or capable of adapting to them. This approach includes domain generalization (C. Xu et al., 2024), domain randomization (Billot et al., 2023), and domain adaptation (Dinsdale et al., 2021; Gebre et al., 2023; Guan et al., 2021; Perone et al., 2019; R. Wang et al., 2022) methods, which are machine learning techniques that enable greater model generalization to accommodate heterogeneity. The second approach to addressing heterogeneity is the application of retrospective harmonization, which refers to methods that transform images or measurements to ensure they appear as though they were acquired using a single, consistent acquisition protocol. Harmonization algorithms, which are the primary focus of this work, offer a significant advantage in that they are flexible with respect to the segmentation algorithm used to perform volumetry. This flexibility allows for a greater choice of algorithms and potentially more specialized measurements, such as those related to cortical morphometry.

Retrospective harmonization can be achieved either by adjusting the measurements derived from acquired images using statistical algorithms, or by first correcting the acquired images and subsequently deriving measurements from the corrected data. Measurement harmonization is typically performed using a statistical correction, such as employing a site covariate within the statistical model during analysis (Chua et al., 2015; Habes et al., 2021; Jones et al., 2013). NeuroCombat is a widely used statistical measurement harmonization approach that removes unwanted batch effects from neuroimaging data collected across multiple acquisition protocols or time points (Bathla et al., 2024; Fortin et al., 2017, 2018; Li et al., 2021). The main advantage of neuroCombat is its ability to effectively harmonize neuroimaging data by adjusting for variations introduced by different sources, such as scanner types, acquisition protocols, or batch effects, without the need for extensive preprocessing or data transformation (Eshaghzadeh Torbati et al., 2021; Han et al., 2023; Radua et al., 2020; Richter et al., 2022). Studies have shown that harmonization of diffusion and positron emission tomography (PET) MRI data is highly recommended, with neuroCombat performing particularly well in both cross-sectional and longitudinal settings (Richter et al., 2022; Zounek et al., 2023). This harmonization method significantly enhances statistical power in neuroimaging studies by reducing site-related heterogeneity, making it especially valuable in large-scale consortium projects (Eshaghzadeh Torbati et al., 2021; Han et al., 2023; Radua et al., 2020). NeuroCombat is more effective than other approaches, such as RAVEL (Eshaghzadeh Torbati et al., 2021) and the RAVEL–ComBat combination (Eshaghzadeh Torbati et al., 2021), in regional-level harmonization due to its more consistent performance across subjects and image-derived measures (Eshaghzadeh Torbati et al., 2021). However, some studies suggest that alternative methods, such as subsampling (Pagani et al., 2023), the Harmonization using Common Orthogonal Basis Extraction (HCOBE) (Zhao et al., 2024), Probabilistic Estimation for Across-batch Compatibility Enhancement (PEACE) (Bilgel, 2023), and the Conditional Variational Autoencoder (cVAE) harmonization model (An et al., 2022), may outperform ComBat in specific scenarios.

Image-based harmonization algorithms perform image-to-image translation to produce images with a single, desired MRI contrast from images with heterogeneous contrast (Guan et al., 2021; Roca et al., 2023; Wrobel et al., 2020). Subsequent volumetric processing can, therefore, proceed as if the data had been collected with the same protocol. Image-based harmonization enhances the generalizability of findings across different populations and settings (Hu et al., 2023). However, a significant drawback is that it may inadvertently remove or distort meaningful biological variability, potentially leading to the loss of critical information related to individual differences (Zhu et al., 2019). Additionally, the harmonization process is often complex, requiring careful selection of appropriate methods, which can introduce challenges in ensuring the reliability and validity of the harmonized data (Mali et al., 2021). While numerous image- and feature-based harmonization methods have been created to mitigate these effects, none have yet been recognized as the most effective for volumetric analysis applications (Hu et al., 2023; Mali et al., 2021; Seoni et al., 2024; Stamoulou et al., 2022).

Despite the improvements in volumetric analysis outcomes attributed to harmonization methodologies, a thorough comparative analysis of their performance and efficiency remains largely unexplored. Gebre et al. (2023) conducted an evaluation of various harmonization techniques, including deep learning approaches (Neural Style Transfer, CycleGAN, and CGAN), histogram matching, and statistical methods (ComBat and LongComBat), to mitigate noise and bias introduced by scanner and protocol variations in MRI biomarkers associated with aging and dementia. They found that while some methods improved agreement in cross-sectional scans, none succeeded in fully harmonizing longitudinal datasets, highlighting the need for further research.

This study evaluates three distinct harmonization methods: neuroCombat, DeepHarmony (Dewey et al., 2019, 2020), and HACA3 (Zuo et al., 2021, 2023). DeepHarmony and HACA3 are both image-based harmonization techniques with the former being a supervised approach requiring paired image data, and the latter being unsupervised. A curated set of T1-weighted MR images acquired with two different protocols from the same subjects was employed as the basis for the comparison. The evaluation criteria were centered around the consistency of volumetric segmentation measurements across the two protocols and the accuracy in detecting simulated atrophy. By comparing these three techniques in a systematic manner, this study aims to provide insights into the relative strengths and limitations of neuroCombat, DeepHarmony, and HACA3 in the context of harmonizing neuroimaging data.

## 2 Methods

### 2.1 Data acquisition

MR data were collected from 39 healthy subjects (age: 36.4 ± 10.0 years, range: 22–64 years, sex: 14M/25F, Supplementary Table S1). Two types of T1-weighted images were acquired for each subject on a Siemens 3T Skyra MRI Scanner (Siemens Medical Solutions Inc., Malvern, PA) within a single scan session: (1) three-dimensional T1-weighted spoiled gradient-recalled echo (GRE, 256 x 256 x 192, repetition time = 7.8 ms, echo time = 3 ms, flip angle = 18°) images and (2) magnetization prepared rapid gradient echo (MPRAGE, 256 x 256 x 176, repetition time = 3000 ms, echo time = 3.03 ms, inversion time = 900 ms, flip angle = 9°) images. The voxel size was 1 × 1 × 1 mm^3^ for both acquisitions.

### 2.2 Image preprocessing

**Fig. 1.**
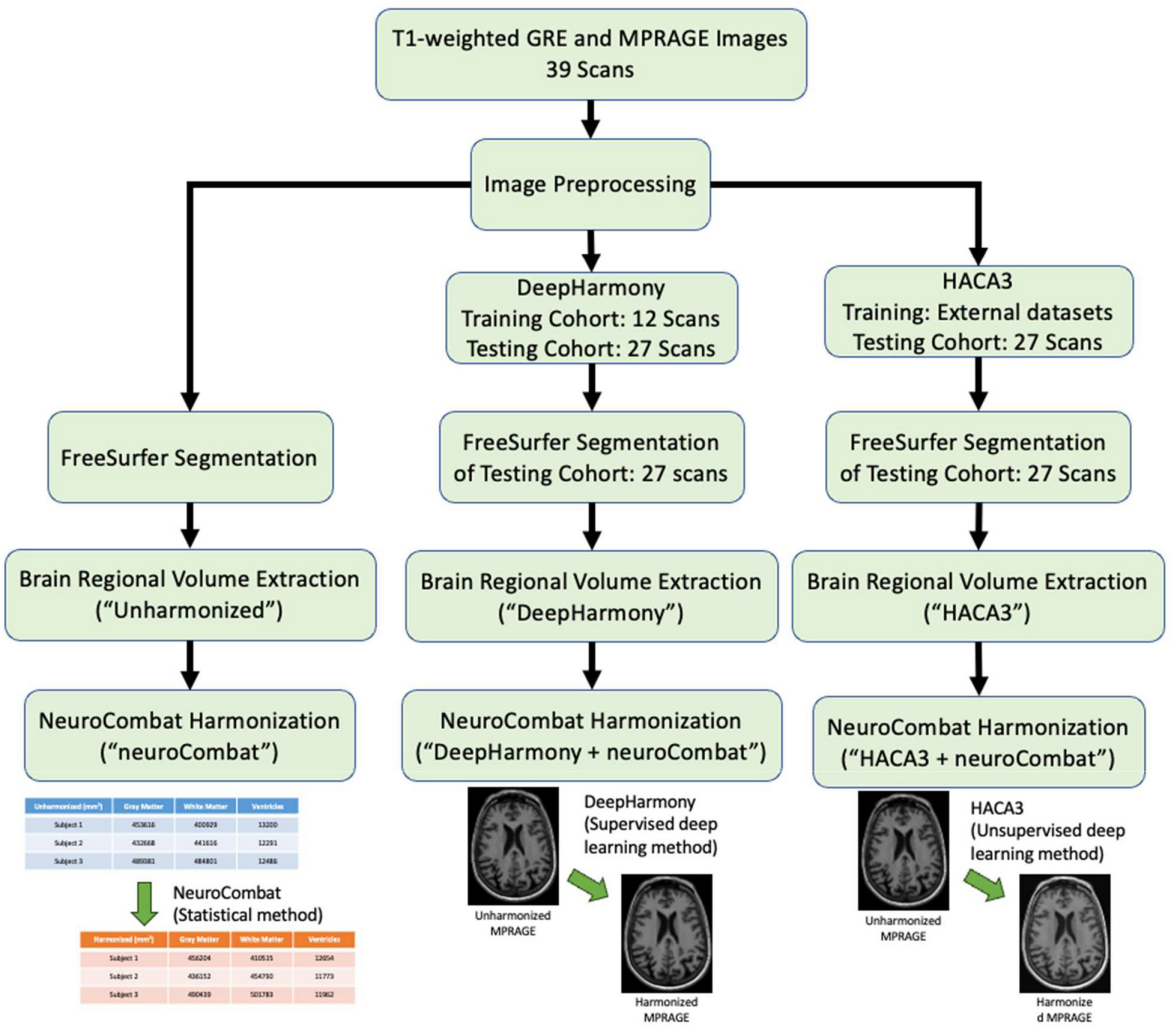
Harmonization procedures using neuroCombat, DeepHarmony, and HACA3 methods.

All images underwent conversion to the Neuroimaging Informatics Technology Initiative (NIfTI-1) file format. Following this conversion step, inhomogeneity correction was applied using N4ITK (Tustison et al., 2010). For each subject, the GRE images underwent rigid registration to the MPRAGE images employing the Advanced Normalization Tools (ANTs) software package (Dewey et al., 2020). To ensure accurate alignment, a two-step rigid registration process was performed. The initial registration involved the entire head image, followed by a subsequent registration utilizing only brain voxels isolated with ROBEX (Iglesias et al., 2011). Following registration, a linear intensity normalization was applied to all images to align the peaks of the white matter histogram (WMPs), as previously described (Reinhold et al., 2019). Briefly, determination of the WMP involved calculating the mean intensity within a preliminary WM mask. These tissue masks were generated on the GRE and MPRAGE images using intensity clustering within brain tissue, isolating regions roughly corresponding to cerebral WM, cortical gray matter (GM), and cerebrospinal fluid. The resulting gain-corrected, co-registered images were divided into a training set (12 subjects) and a testing set (27 subjects), which served as inputs for the harmonization process (Fig. 1). The unharmonized images were also used as a basis for comparison with the harmonized results.

### 2.3 NeuroCombat

NeuroCombat employs a linear regression model to estimate and adjust for “site-specific” effects while preserving the underlying biological signal, ensuring the harmonized data remain suitable for subsequent analyses. In this study, we applied the publicly available neuroCombat R package (R version 4.2.2) to harmonize volume measurements between the acquired GRE and MPRAGE images, treating the two imaging contrasts as distinct sites (Fortin et al., 2017, 2018). Some studies have applied neuroCombat without specified training sets for harmonizing brain regional volumes, cortical thickness (Richter et al., 2022), and magnetic resonance spectroscopy data (Bell et al., 2022), while others used trained models, where the regression parameters are estimated on a separate dataset, to harmonize testing set features (Marzi et al., 2024; Stamoulou et al., 2021). Our goal was to assess the harmonization performance of neuroCombat both with and without trained models. NeuroCombat can be applied directly to a dataset without the requirement of a separate training model, making it straightforward to use for harmonizing data with batch or site effects. However, the use of training models is recommended when the goal is to apply the same harmonization parameters to future datasets, ensuring consistency across studies or longitudinal data collections. We refer to the trained version of neuroCombat as CombatT and the untrained version as CombatU. For CombatT, the training set used brain regional volumes (referred to as “features” in the neuroCombat nomenclature) measured from two acquisition types in 12 subjects. Subsequently, the trained models were applied to the testing set for harmonization. A numeric vector denotes the “batch,” indicating the two types of images (0 for GRE and 1 for MPRAGE). No biological covariates were included in any of the neuroCombat models utilized in this study.

This study compared three neuroCombat options: one without empirical Bayes (EB), one with EB and parametric priors on the batch effect parameters (“parametric adjustments” or PA), and one with EB and non-parametric priors on the batch effect parameters (“without parametric adjustments” or without PA). In the EB method with PA, prior distributions—specifically, normal distribution and inverse gamma distribution—were assumed for the batch effect parameters (γ and $\delta^{2}$, respectively) (Fortin et al., 2017, 2018). These distributions were estimated empirically from the data. NeuroCombat without EB is recommended when the prior distributions used in neuroCombat do not align well with the data (Fortin et al., 2017, 2018).

### 2.4 DeepHarmony

DeepHarmony is a supervised image-based harmonization approach designed to address domain shift in MRI data when paired images are available. This method leverages deep learning techniques to adjust image appearances while preserving their underlying anatomical information (Dewey et al., 2019, 2020). It uses a two-dimensional U-Net architecture to perform image-to-image translation, aligning their intensity, noise, and contrast characteristics, making them suitable for consistent analysis (Dewey et al., 2019).

**Fig. 2.**
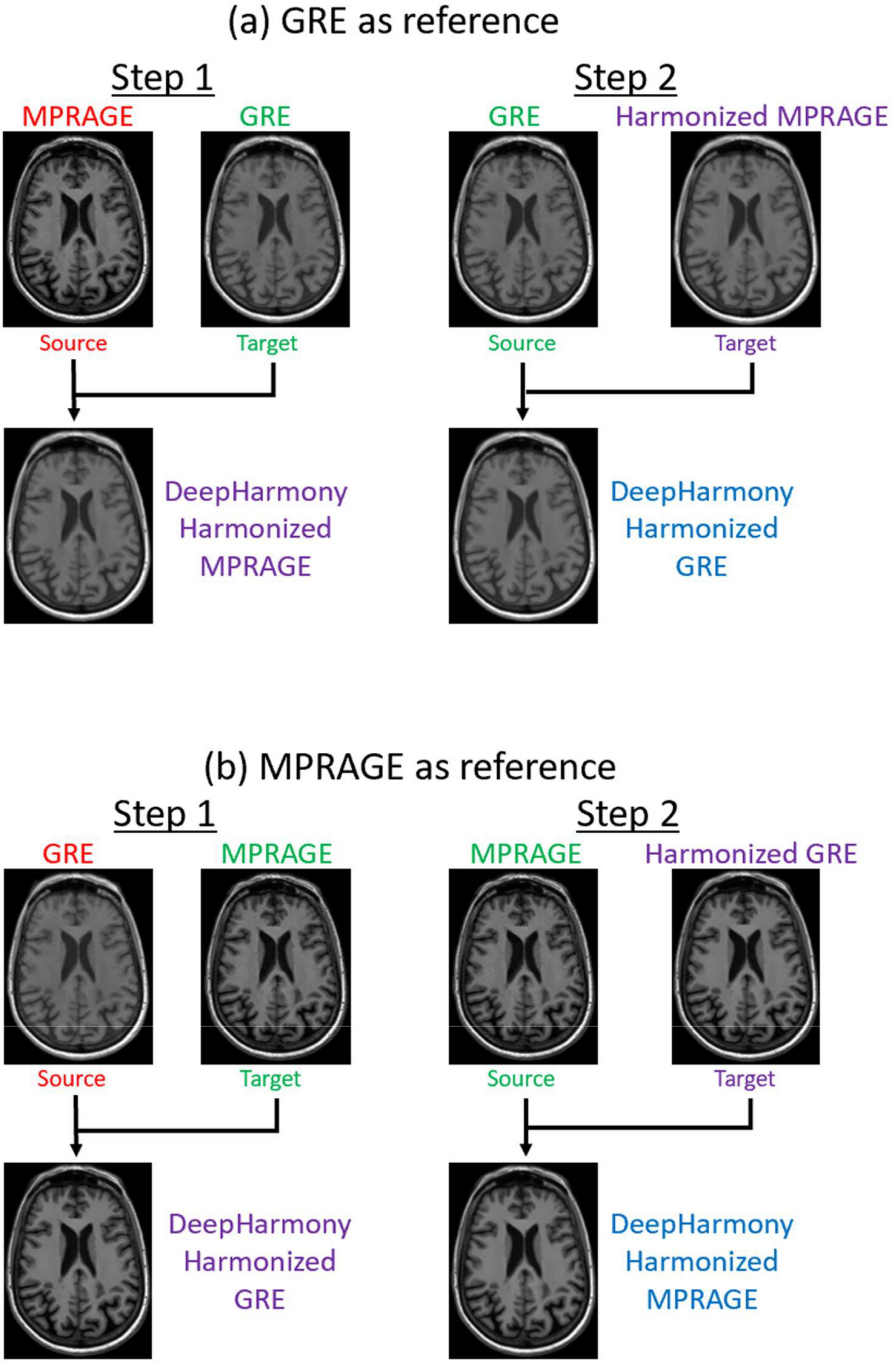
Example results after training the neural network models of GRE and MPRAGE synthesis using DeepHarmony. (a) GRE as the target contrast. (b) MPRAGE as the target contrast. Note that DeepHarmony includes a step (“Step 2”) to synthesize the target contrast even if the input and target contrasts are the same for increased consistency.

Two groups of neural network models were trained using the training cohort: (1) paired MPRAGE images as “source” and the GRE images as “target” (Fig. 2a) and (2) paired GRE images as “source” and MPRAGE images as “target” (Fig. 2b). In each group, the harmonization process involved two steps. First, three models were trained to harmonize the source contrast to the target contrast in three different orientations (axial, coronal, and sagittal planes). During testing, the outputs of these three models were combined using the median value at each voxel. Second, three models were trained to harmonize the target contrast to “itself” by training on the harmonized output images from the first step as the target in the same three orientations. The median value was again used to combine the output of the three models. As a result of these two steps, DeepHarmony processes all images, not just the images with the source contrast, thereby limiting domain shift due to different noise characteristics in the original and processed images that might affect the subsequent segmentation.

### 2.5 HACA3

Harmonization with Attention-based Contrast, Anatomy, and Artifact Awareness (HACA3) is an unsupervised image synthesis-based harmonization approach designed to work with a single or multiple structural MRI contrasts (Zuo et al., 2021, 2023). This method is designed to accommodate various MR contrasts and is not limited to T1-weighted images. It differs from neuroCombat’s statistical harmonization by operating directly in the image space, so it avoids making assumptions about prior variations of data. Unlike DeepHarmony, which requires paired data from the GRE and MPRAGE cohorts for training, HACA3 is trained using MRI data with different contrast images that can be from different subjects. This distinction of HACA3 allows for broader applicability across various imaging cohorts with improved flexibility and generalizability.

**Fig. 3.**
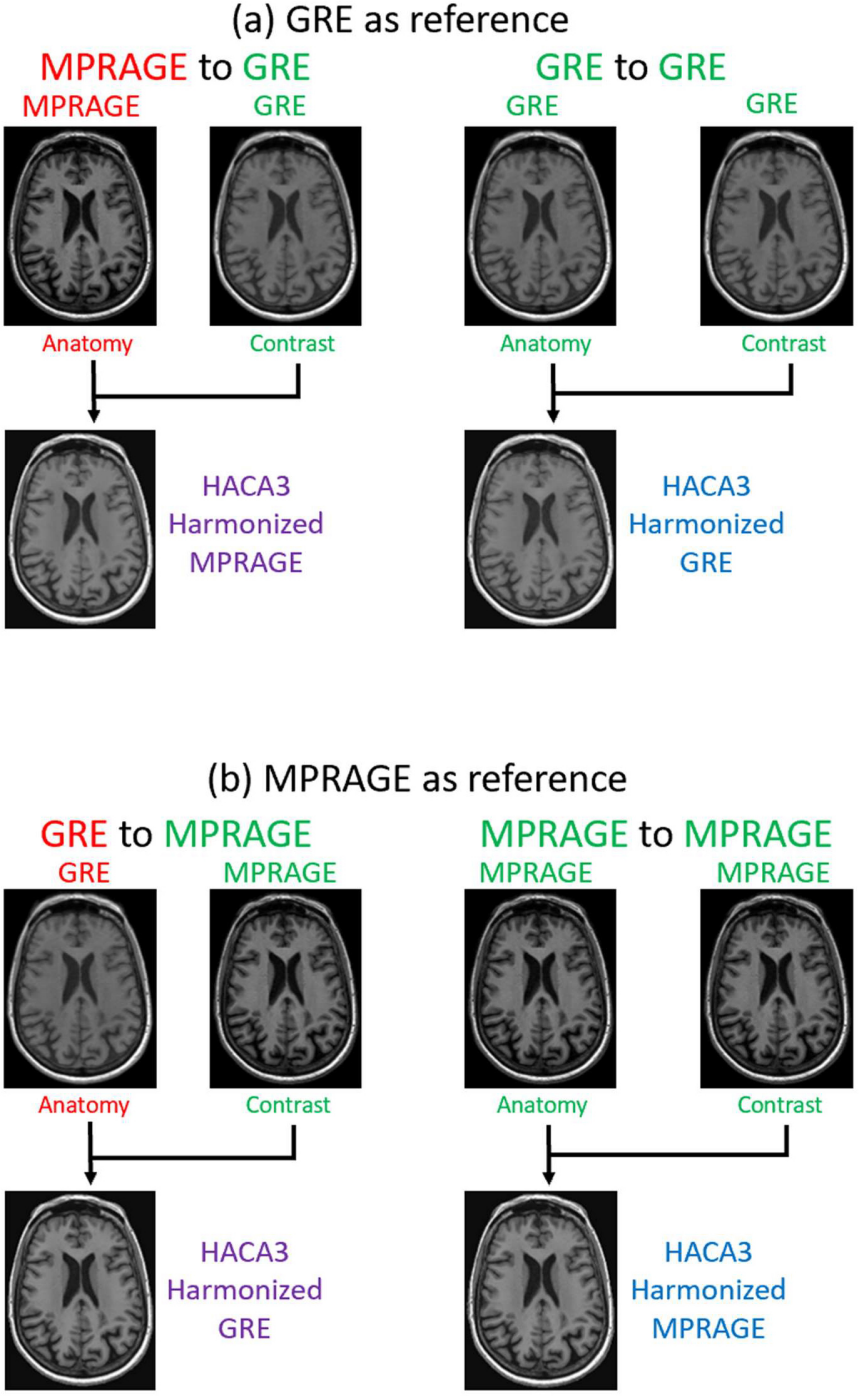
Example results after training the neural network models of GRE and MPRAGE synthesis using HACA3. (a) GRE as the target contrast. (b) MPRAGE as the target contrast.

The core of HACA3 is an autoencoder-like framework, which consists of an encoding and a decoding step (Zuo et al., 2023). During encoding, HACA3 learns latent representations of anatomy, contrast (i.e., acquisition related), and image quality. The decoding phase involves reassembling these latent representations to create a synthetic MR image. Once trained, HACA3 harmonizes MR images across different cohorts by extracting the anatomy representation from a source cohort and combining it with the contrast representation of a target cohort image (Fig. 3). The harmonized image maintains the anatomy information from the source image while adopting the desired contrast information of the target image.

In our study, HACA3 was trained on a diverse set of 21 external imaging datasets (table 1 in Zuo et al., 2023) that did not include the GRE and MPRAGE cohorts utilized in our analysis. The datasets used to train HACA3 were preprocessed using the same pipeline described in Section 2.2 to ensure consistency across datasets, including white matter peak normalization. The HACA3 training datasets included multiple MR contrasts, such as T1-weighted, T2-weighted, Proton Density-weighted (PD-w), and Fluid-Attenuated Inversion Recovery (FLAIR) images, acquired using different scanning hardware, pulse sequences, and imaging parameters. Similar to the experiment using DeepHarmony, we first chose the GRE cohort as our target cohort and harmonized images from both GRE and MPRAGE cohorts to the target GRE cohort, as shown in Figure 3a. Similarly, we then chose the MPRAGE cohort as our target cohort, as we show in Figure 3b. All output images were synthetic in both scenarios. Notably, due to HACA3’s unsupervised nature, the target image in these experiments was chosen randomly from the testing set and did not need to match the source image anatomy. This step runs the target image through the encoder to obtain the latent space parameters pertaining to the contrast, and it was used to harmonize all images from a source domain (anatomical latent space) to that target domain. Subsequently, we compared the brain regional volumes between the cross-cohort harmonized images and self-harmonized images to observe the volume differences. We aim to assess the effectiveness of HACA3 in reducing inconsistencies caused by acquisition from different imaging cohorts.

**Table 1.** Absolute volume difference percentages (AVDP, %, mean ± standard deviation) of regional brain volumes between GRE and MPRAGE images of the testing cohort (N = 27) for unharmonized data, harmonized by neuroCombat with trained models (CombatT), and harmonized by neuroCombat without trained models (CombatU).

		CombatT			CombatU		
Brain regions	Unharmonized	with EB and without PA	with EB and with PA	without EB	with EB and without PA	with EB and with PA	without EB
Whole brain	4.22 ± 1.31	2.78 ± 1.24*^#^	1.62 ± 1.10*	1.14 ± 1.18*	2.81 ± 1.19*^#^	1.41 ± 0.79*	**1.14** **±** 0.82*
Ventricles	8.66 ± 3.63	11.23 ± 4.48*^#^	6.91 ± 2.96*^#^	2.74 ± 2.75*	4.78 ± 2.75*^#^	3.50 ± 2.20*^#^	**1.97** **±** 2.15*
Cortical GM	2.22 ± 1.59	**2.02** **±** **1.28**	2.08 ± 1.47	2.06 ± 1.45	2.16 ± 1.58	2.10 ± 1.39	2.08 ± 1.33
Cerebral WM	6.49 ± 3.52	5.01 ± 3.17*^#^	3.36 ± 2.89*	3.35 ± 2.91*	4.33 ± 2.94*^#^	2.93 ± 2.16*	**2.84** **±** 1.55*
Cerebellar GM	7.94 ± 4.27	7.38 ± 3.84*	7.13 ± 3.98	7.06 ± 4.27	7.32 ± 3.97	6.96 ± 4.38	**6.66** **±** **4.68**
Cerebellar WM	31.68 ± 24.67	25.28 ± 22.09*	21.90 ± 17.09*	24.09 ± 18.83	25.34 ± 21.42*	**21.48** **±** 15.20*	23.41 ± 17.80
Brainstem	5.12 ± 2.71	4.81 ± 2.65*	4.86 ± 2.63*	**4.50** **±** **2.83**	5.19 ± 2.46	4.97 ± 2.51	4.65 ± 2.71
Thalamus	5.72 ± 3.56	4.43 ± 3.01*	4.01 ± 3.02*	3.70 ± 3.40	5.00 ± 3.10*^#^	3.95 ± 2.97*	**3.66** **±** 2.96*
Caudate	4.79 ± 4.08	4.15 ± 3.40*	3.78 ± 2.85*	3.98 ± 2.50	4.05 ± 3.25*	3.88 ± 2.83	**3.69** **±** **2.61**
Putamen	14.34 ± 6.91	11.85 ± 6.12*^#^	8.74 ± 5.50*	**6.58** **±** 6.19*	11.38 ± 5.96*^#^	8.01 ± 5.22*	6.83 ± 5.14*
Hippocampus	3.55 ± 2.87	3.54 ± 2.88	3.67 ± 3.08	4.12 ± 3.09	3.45 ± 2.79	3.34 ± 3.02	**3.32** **±** **2.93**
Amygdala	9.08 ± 5.37	8.67 ± 5.29*^#^	7.44 ± 5.48*	6.31 ± 5.81*	7.92 ± 5.51*^#^	6.92 ± 5.77*	**6.30** **±** 6.08*
Average	9.05 ± 2.41	8.03 ± 2.42*^#^	6.72 ± 2.33*^#^	6.23 ± 2.42*	7.36 ± 2.30*^#^	6.19 ± 2.10*	**5.95** **±** 2.25*

* : *p_adj_* < 0.05 for paired t-tests of AVDP between the **unharmonized data** versus neuroCombat scenarios. #: *p_adj_* < 0.05 for paired t-tests of AVDP between **CombatU without empirical Bayes** versus other neuroCombat scenarios. The averages of the AVDP values for the 11 brain regions, along with the AVDP values for the whole brain, are presented and compared with the unharmonized data (*: *p-value* < 0.05) and with CombatU without empirical Bayes (#: *p-value* < 0.05). Bold: Lowest mean value of AVDP among the compared scenarios for each brain region and the whole brain.

### 2.6 Brain segmentation and volume comparison

#### 2.6.1 FreeSurfer

**Fig. 4.**
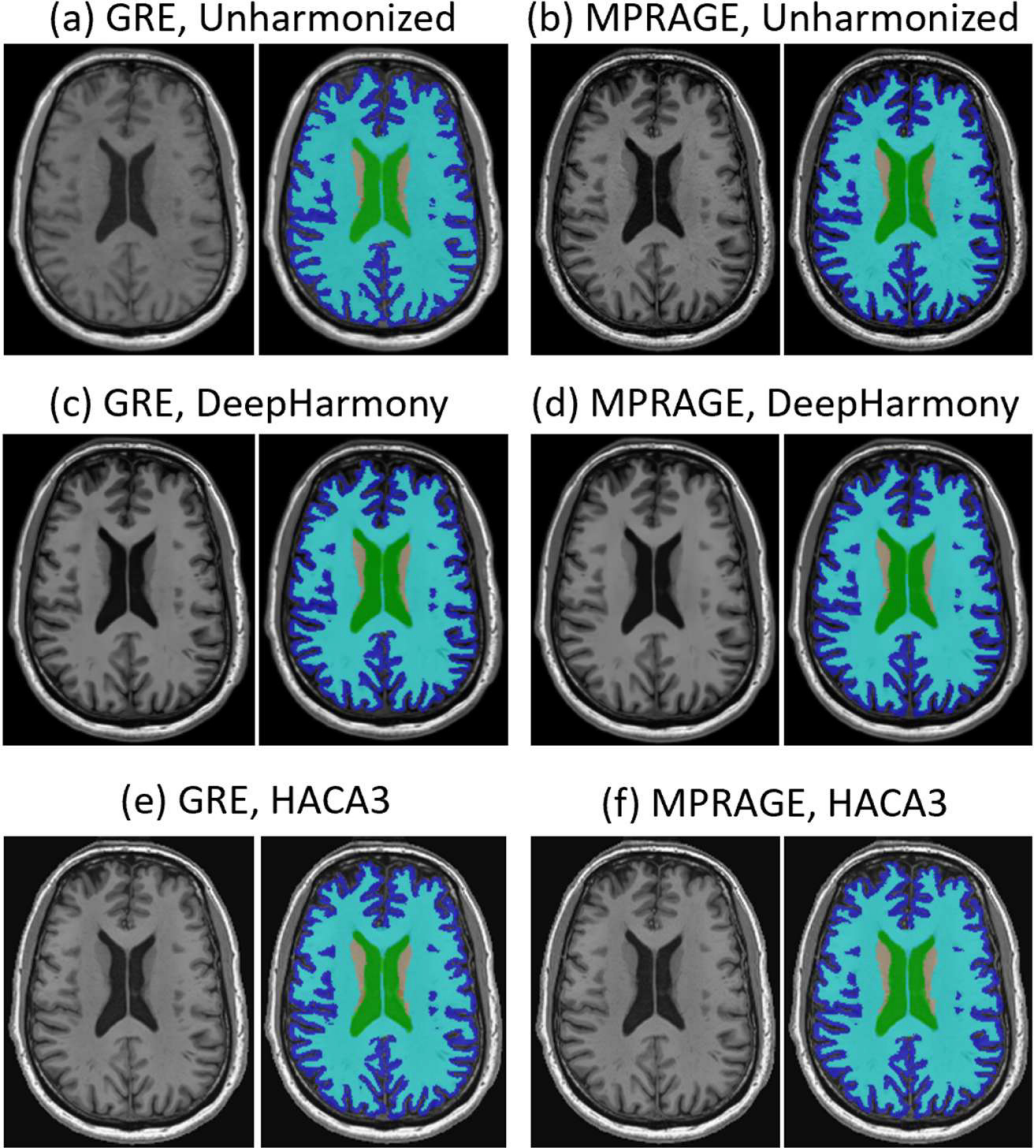
Image segmentation by FreeSurfer. (a) and (b): Unharmonized images; (c) and (d): harmonized by DeepHarmony (with MPRAGE as reference); (e) and (f): harmonized by HACA3 (with MPRAGE as reference).

Whole brain segmentation was applied to the unharmonized and harmonized images using FreeSurfer version 7.2.0, and selected volumes of 11 brain regions were calculated (Fig. 4). These brain regions included bilateral ventricles, cortical GM, cerebral WM, cerebellar GM, cerebellar WM, brainstem, thalamus, caudate, putamen, hippocampus, and amygdala. The volume difference percentage (VDP) and its absolute value (AVDP) between the corresponding brain regional volumes determined from the GRE and MPRAGE images were calculated using the following equations (Guo et al., 2019).

$${\text{VDP}}_{i}=\frac{{\text{Volume}}_{\text{GRE},i}-{\text{Volume}}_{\text{MPRAGE},i}}{\left({\text{Volume}}_{\text{GRE},i}+{\text{Volume}}_{\text{MPRAGE},i}\right)\;/\text{​}2}\times 100\text{\%}$$ (1)

$${\text{AVDP}}_{i}=\;\left|{\text{VDP}}_{i}\right|,$$ (2)

where i=1,…,11 , representing the 11 brain regions. Unless specified (see Section 2.6.2), the segmentation results of brain regional volumes presented in this study were conducted using FreeSurfer. VDP and ADVP were calculated exclusively on the testing dataset. The selection of the optimal model among the compared scenarios was based on the consistency of brain regional volumes between the two types of T1-weighted images using the AVDP metric with paired two-sample t-tests (see Section 2.8.1). For neuroCombat, the unharmonized data were first compared with all neuroCombat scenarios, followed by a comparison of CombatU without EB against other neuroCombat scenarios, including CombatT with and without EB, as well as CombatU with EB. For DeepHarmony and HACA3, the AVDPs calculated from GRE as reference and MPRAGE as reference were compared. A lower AVDP reflects a higher agreement between the two volume measurements, suggesting a more effective model in this evaluation.

#### 2.6.2 SynthSeg

While FreeSurfer’s strength lies in its ability to generate high-resolution surface models and volumetric segmentations, it does not directly address variations in MRI contrast. We, therefore, also used SynthSeg, an alternative segmentation algorithm included in the FreeSurfer software package, which is designed to be robust to contrast variation. SynthSeg, a deep learning-based approach, employs a domain randomization strategy to provide diverse contrast and resolution properties in a synthetic training dataset for brain segmentation. As a result, SynthSeg is capable of segmenting MR scans across various target domains without the need for retraining or fine-tuning. The mri_synthseg tool in SynthSeg was utilized to segment brain structures for unharmonized GRE and MPRAGE images.

### 2.7 Sensitivity analysis

**Fig. 5.**
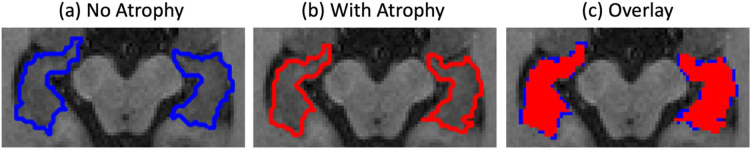
Illustration of hippocampal atrophy in a subject from the testing cohort. (a) FreeSurfer contour of the hippocampus without atrophy. (b) Contour of the hippocampus with atrophy. (c) Overlay of hippocampal segmentation. Blue: without atrophy. Red: with atrophy.

To examine whether the three harmonization approaches were able to detect subtle changes in regional brain structures despite changes in contrast, we simulated brain atrophy around the hippocampus (Fig. 5). This scenario mimics a longitudinal MRI study where the protocol is changed between the first and second time point. We assumed that the first time point was acquired using a GRE acquisition while the second time point was acquired using an MPRAGE acquisition, following atrophy primarily around the hippocampus. The simulation of atrophy involved the nonlinear transformation of brain structures of the brain images for the MPRAGE testing set. We first utilized the FreeSurfer tool (version 7.2.0) mri_label2vol to convert the segmented anatomical labels from the FreeSurfer output into a volumetric representation. In particular, the label mask for the hippocampus was selected to be altered because its atrophy occurs in aging and a number of neurological disorders (McEwen, 1997; Sengoku, 2020). Binary erosion of the bilateral hippocampal mask with a kernel radius of 1 voxel was performed to simulate localized atrophy. Once we obtained a binary mask for the atrophy result, we next applied deformable registration of the original mask to the eroded mask using the SyN algorithm in ANTs. Finally, we applied 25% of the deformation field to the preprocessed images yielding a set of two time points with known changes in the images. Simulated atrophy images were harmonized using neuroCombat, DeepHarmony, and HACA3, and then compared with preprocessed MPRAGE images without atrophy as the reference standard, as well as to preprocessed GRE images without atrophy.

### 2.8 Statistical analysis

#### 2.8.1 Paired two-sample t-tests

AVDPs between each pair of GRE and MPRAGE brain volumes in the unharmonized data and the three harmonization approaches (neuroCombat, DeepHarmony, and HACA3) were compared using paired two-sample t-tests to evaluate the mean differences. The adjusted *p*-values were determined through multiple comparisons using the Benjamini–Hochberg method, taking into account the number of brain regions, controlling the false discovery rate at 5% (Haynes, 2013). Within the Benjamini–Hochberg method, hypotheses were initially arranged in order, and their acceptance or rejection was subsequently determined based on their respective *p*-values.

#### 2.8.2 Median deviation and variance equality

Deviations from zero in the VDPs were analyzed using the Wilcoxon Signed-Rank test (Armstrong et al., 2011). Variance equality between the unharmonized and each harmonized dataset was evaluated using the Brown–Forsythe test, a variation of Levene’s test that employs the median instead of the mean (Armstrong et al., 2011). Adjusted p-values were calculated using the Benjamini–Hochberg method to account for multiple comparisons across brain regions, controlling the false discovery rate at 5%.

#### 2.8.3 Intra-class correlation

The intra-class correlation (ICC) measurements between GRE and MPRAGE images for the unharmonized and harmonized data (neuroCombat, DeepHarmony, and HACA3) were also determined (McGraw & Wong, 1996). ICC assesses the reliability and agreement between measurements or ratings made by multiple observers or methods on the same set of subjects. ICC quantifies the proportion of total variance attributable to true differences between subjects relative to the total variance, encompassing both true differences and measurement error. The calculation involves the division of the between-subject variance by the sum of the between-subject and within-subject variances. Higher values indicate better agreement between the two imaging measurements. Consistency is considered poor for values below 0.5, moderate for values ranging between 0.5 and 0.75, good for values between 0.75 and 0.9, and excellent for values exceeding 0.90.

#### 2.8.4 Coefficient of variation

For each brain region, the coefficient of variation (CV) was calculated correspondingly for the GRE and MPRAGE images for the unharmonized and harmonized data (neuroCombat, DeepHarmony, and HACA3) (Sokal & Braumann, 1980). CV expresses the relative variability of a dataset by quantifying the ratio of the standard deviation to the mean, typically expressed as a percentage. Sokal and Braumann introduced an adjustment to the traditional CV to address concerns related to its sensitivity to extreme values (Eq. 3) (Sokal & Braumann, 1980),

$${\text{CV}}^{*}=\left(1+\frac{1}{4n}\right)\frac{s}{\bar{x}},$$ (3)

where n is the sample size, s is the sample standard deviation, and $\bar{x}$ is the sample mean. This adjusted CV, denoted as CV*, incorporates a correction factor based on the absolute deviation of each data point from the median. This modification aimed to enhance the robustness of the CV in the presence of outliers, making it a more reliable measure of relative variability.

Bland–Altman plots were employed to assess the agreement between CV values derived from GRE and MPRAGE images. The plots illustrate the differences between the CV measurements for GRE and MPRAGE images against the average of these measurements, for both unharmonized and harmonized datasets (neuroCombat, DeepHarmony, and HACA3). The average of CV measurements between the GRE and MPRAGE images was defined as

$${\text{Average of CV}}_{i}=\frac{{\text{CV}}_{\text{GRE},i}+{\text{CV}}_{\text{MPRAGE},i}}{2},$$ (4)

where i=1,…,11,  representing the 11 brain regions, while the difference of CV measurements between the GRE and MPRAGE images was defined as

$${\text{Difference of CV}}_{i}={\text{CV}}_{\text{GRE},i}-{\text{CV}}_{\text{MPRAGE},i}.$$ (5)

The mean difference and the 95% confidence intervals, calculated as the mean difference plus and minus 1.96 times the standard deviation of the differences, were key indicators of agreement.

### 2.9 Combination of harmonization methods

The integration of DeepHarmony+neuroCombat and HACA3 + neuroCombat was implemented to assess whether neuroCombat, as an additional step, could further refine the corrections achieved by DeepHarmony and HACA3. Because these deep learning approaches do not model downstream measurements, they may not fully account for volumetric variations. NeuroCombat, designed to remove batch effects while preserving biological variability, may help mitigate residual systematic differences in the volume measurement between imaging modalities. By applying neuroCombat after DeepHarmony and HACA3, we aimed to evaluate whether harmonization consistency could be further improved, reducing bias, and enhancing statistical comparability. The resulting harmonized brain regional volumes using the combined DeepHarmony–neuroCombat or HACA3–neuroCombat methods were compared with DeepHarmony or HACA3 method alone, correspondingly, using the AVDP and ICC.

## 3 Results

### 3.1 Consistency analysis

#### 3.1.1 Selection of neuroCombat models

A comparative analysis of brain regional volumes between GRE and MPRAGE images was conducted for CombatT, CombatU, and the three options of EB without PA, EB with PA, and without EB. Supplementary Figure S1 shows the comparison of volume measurements, while Table 1 shows the AVDP between the two measurements. Asterisks in Supplementary Figure S1 indicate statistically significant differences between the GRE and MPRAGE measurements. It is evident that, without harmonization, significant differences were observed in 8 out of the 11 volume measurements. CombatT performed best without EB, with only significant differences in ventricle volume following harmonization. In contrast, all other CombatT harmonization approaches resulted in multiple brain structures (e.g., cerebral WM, cerebellar WM, ventricles, and putamen) showing significant differences in volume measurements between GRE and MPRAGE images. Similarly, CombatU performed best without EB, showing no significant differences in volume measurements between GRE and MPRAGE images, compared with CombatU with EB.

When comparing the AVDP between CombatU without EB versus unharmonized data, the AVDP was significantly reduced in the ventricles, cerebral WM, thalamus, putamen, and amygdala (*p_adj_* < 0.05) (Table 1). Additionally, when comparing CombatU with EB methods to CombatU without EB, the AVDPs were significantly higher in some brain regions, including the ventricles, cerebral WM, thalamus, putamen, and amygdala (*p_adj_* < 0.05). However, no significant differences in AVDP were identified between CombatT and CombatU when EB was not used. This implies that neuroCombat without EB was the more effective approach for harmonizing our GRE and MPRAGE data compared with neuroCombat with EB methods. We, therefore, did not use EB in the results of the remainder of the paper.

#### 3.1.2 Selection of DeepHarmony target contrast

Brain regional volumes were compared between GRE and MPRAGE images under the conditions of DeepHarmony with GRE as the target contrast and with MPRAGE as the target (Supplementary Fig. S2). When MPRAGE was used as the reference, there were no statistically significant differences in brain regional volumes between GRE and MPRAGE images (*p_adj_* > 0.05). In contrast, using GRE as a reference, a significant difference was observed in the volume of the putamen (*p_adj_* < 0.05). These results suggest that MPRAGE as reference may provide more consistent volumetric measurements across the regions examined. When comparing the AVDPs of regional brain volumes between DeepHarmony with GRE as reference and with MPRAGE as reference, AVDP was significantly reduced for cortical GM, cerebral WM, cerebellar GM, cerebellar WM, and brainstem (*p_adj_* < 0.05) (Table 2), also indicating that MPRAGE as the reference in DeepHarmony could be the more reliable technique for comparative brain volume studies. Therefore, MPRAGE as the DeepHarmony reference was used in the analysis that follows.

**Table 2.** Absolute volume difference percentages (AVDP, %, mean ± standard deviation) of regional brain volumes between GRE and MPRAGE images of the testing cohort (N = 27), harmonized by DeepHarmony.

Brain regions	GRE as reference	MPRAGE as reference
Whole brain	1.20 ± 1.30	**0.73** **±** **0.67**
Ventricles	2.09 ± 1.71	**1.87** **±** **1.46**
Cortical GM	3.67 ± 2.55	**1.35** **±** 1.18*
Cerebral WM	4.79 ± 4.27	**1.57** **±** 1.11*
Cerebellar GM	5.79 ± 6.14	**1.54** **±** 0.87*
Cerebellar WM	17.95 ± 17.29	**6.46** **±** 6.08*
Brainstem	**2.78** **±** **2.18**	6.63 ± 4.37*
Thalamus	**4.64** **±** **2.94**	4.72 ± 5.70
Caudate	**2.14** **±** **1.73**	2.35 ± 2.02
Putamen	5.54 ± 3.40	**5.13** **±** **5.56**
Hippocampus	3.13 ± 2.36	**2.60** **±** **1.70**
Amygdala	**5.14** **±** **4.25**	5.20 ± 4.28
Average	5.24 ± 2.09	**3.58** **±** 1.59*

* : *p_adj_* < 0.05 for paired t-tests of AVDP conducted between DeepHarmony with GRE as reference and DeepHarmony with MPRAGE as reference. The averages of the AVDP values for the 11 brain regions, along with the AVDP values for the whole brain, are presented and compared between DeepHarmony with GRE as reference and DeepHarmony with MPRAGE as reference (*: *p-value* < 0.05). Bold: Lowest mean value of AVDP among the two scenarios for each brain region and the whole brain.

#### 3.1.3 Selection of HACA3 target contrast

A side-by-side comparison of brain regional volumes between GRE and MPRAGE images was conducted using each sequence alternately as a reference contrast for HACA3 (Supplementary Fig. S3). When using GRE as the reference, significant differences in volumes were detected in cortical GM, ventricles, thalamus, and hippocampus. However, fewer differences were found using MPRAGE as the reference, with only cortical GM, cerebral WM, and ventricles showing statistical significance. When comparing the AVDPs between HACA3 with GRE as reference and HACA3 with MPRAGE as reference, the AVDPs were significantly reduced in ventricles, cortical GM, cerebral WM, cerebellar GM, cerebellar WM, brainstem, caudate, and putamen (*p_adj_* < 0.05) (Table 3). Similar to the DeepHarmony results, these findings suggest that MPRAGE may be a more stable reference than GRE for volumetric studies of the brain when using HACA3, implying a higher degree of reliability or consistency in the volumetric measurements provided by the MPRAGE sequence. Therefore, MPRAGE was used as the reference for the remainder of the analysis with the HACA3 method.

**Table 3.** Absolute volume difference percentages (AVDP, %, mean ± standard deviation) of regional brain volumes between GRE and MPRAGE images of the testing cohort (N = 27), harmonized by HACA3.

Brain regions	GRE as reference	MPRAGE as reference
Whole brain	1.34 ± 0.54	**0.58** **±** 0.39*
Ventricles	1.62 ± 1.48	**0.76** **±** 0.61*
Cortical GM	3.42 ± 1.97	**1.61** **±** 0.74*
Cerebral WM	1.68 ± 1.34	**0.66** **±** 0.43*
Cerebellar GM	4.78 ± 4.82	**0.65** **±** 0.54*
Cerebellar WM	15.78 ± 16.32	**2.70** **±** 2.00*
Brainstem	3.08 ± 2.71	**0.82** **±** 0.56*
Thalamus	2.34 ± 1.86	**2.22** **±** **1.94**
Caudate	4.24 ± 4.79	**1.11** **±** 0.74*
Putamen	4.22 ± 4.39	**1.87** **±** 1.83*
Hippocampus	2.14 ± 1.84	**1.32** **±** **1.05**
Amygdala	3.86 ± 3.07	**2.49** **±** **2.31**
Average	4.29 ± 2.02	**1.47** **±** 0.45*

* : *p_adj_* < 0.05 for paired t-tests of AVDP conducted between HACA3 with GRE as reference and HACA3 with MPRAGE as reference. The averages of the AVDP values for the 11 brain regions, along with the AVDP values for the whole brain, are presented and compared between HACA3 with GRE as reference and HACA3 with MPRAGE as reference (*: *p-value* < 0.05). Bold: Lowest mean value of AVDP among the two scenarios for each brain region and the whole brain.

#### 3.1.4 Volume difference percentage

**Fig. 6.**
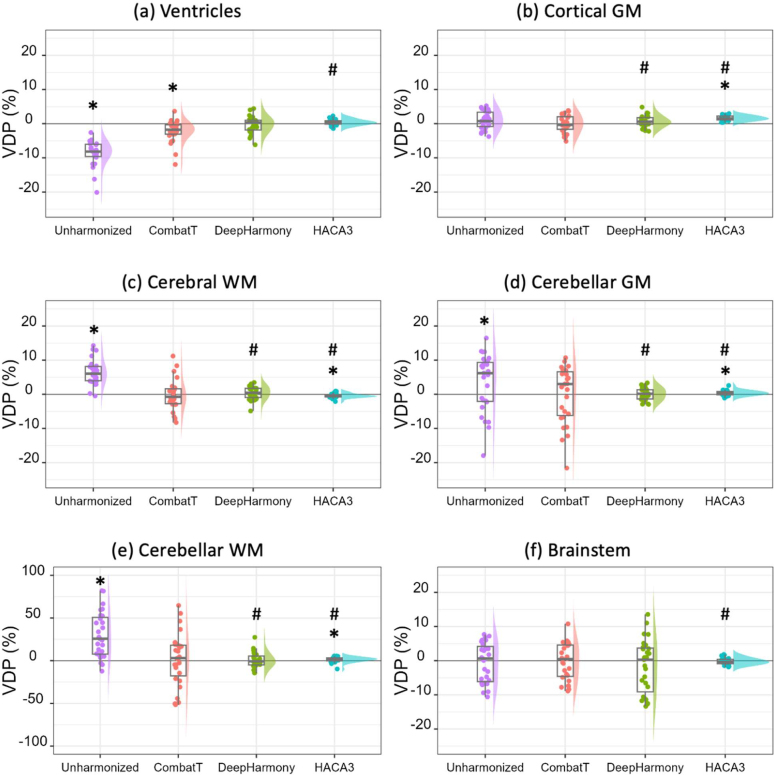

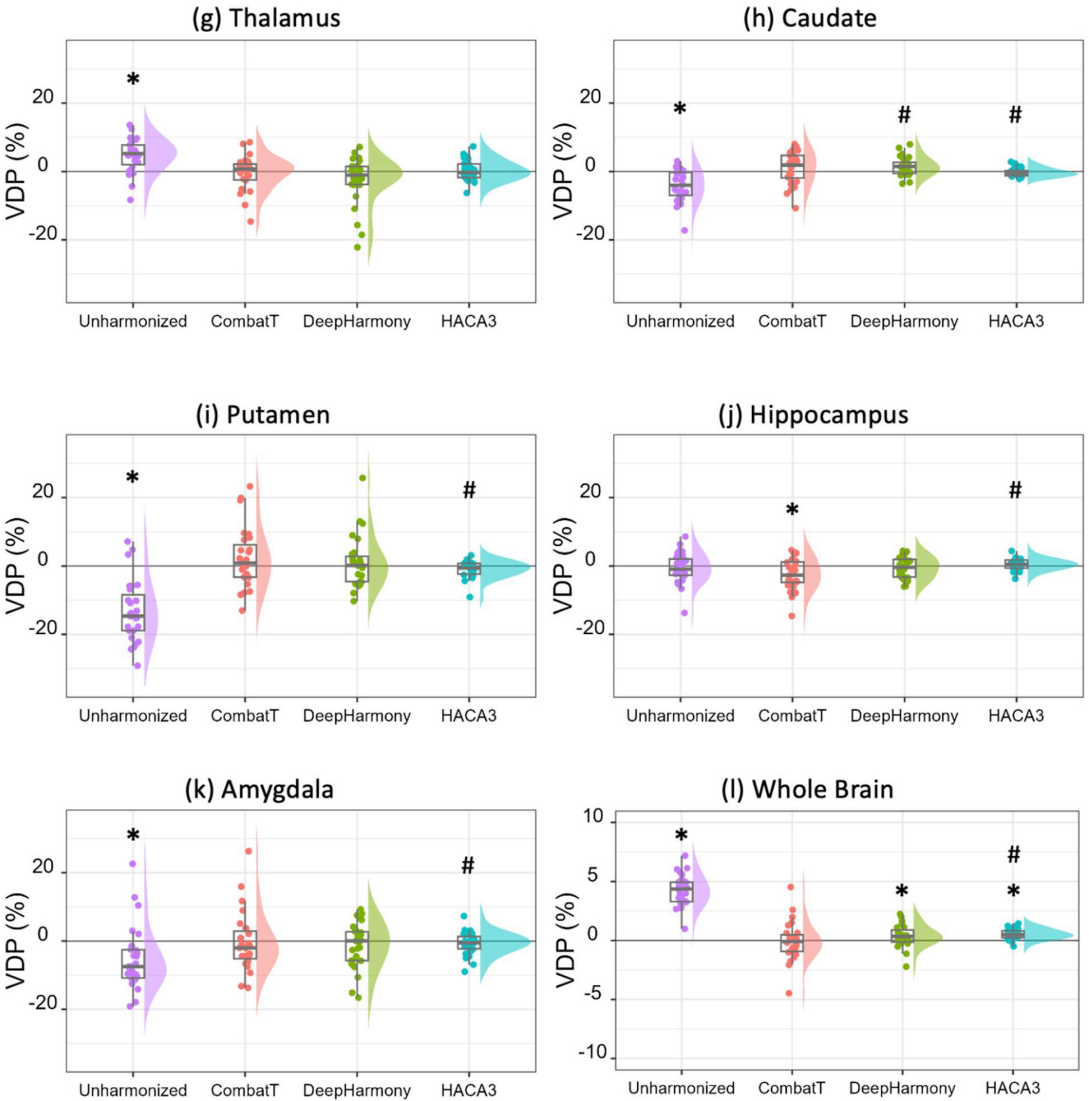
Raincloud plots of the volume difference percentage (VDP, %) of the testing cohort (N = 27) for (a) ventricle; (b) cortical GM; (c) cerebral WM; (d) cerebellar GM; (e) cerebellar WM; (f) brainstem; (g) thalamus; (h) caudate; (i) putamen; (j) hippocampus; (k) amygdala; (l) whole brain. *: *p_adj_* < 0.05 for testing median deviations from zero. #: *p_adj_* < 0.05 for testing variance equality between the unharmonized and each harmonized dataset.

The VDPs between GRE and MPRAGE images were determined for unharmonized data and data harmonized using neuroCombat (CombatT without EB), DeepHarmony (with MPRAGE as reference), and HACA3 (with MPRAGE as reference) and are presented by raincloud plots in Figure 6. A raincloud plot combines a density plot, box plot, and scatter plot to provide a comprehensive view of the distribution, central tendency, and variability of the data (Allen et al., 2019). Ideally, since the images represent the same anatomy, the volume measurements would be identical from the two types of images. The unharmonized data showed considerable variability, which was significantly reduced by each harmonization technique to varying degrees. HACA3 demonstrates the least variation in VDP across all brain regions among the harmonization approaches but exhibited a small but significant positive bias for cortical GM, cerebellar GM, and cerebellar WM and negative bias for cerebral WM (Fig. 6b–e). NeuroCombat did not reduce the measurement variation compared with unharmonized results but effectively removed the bias except for the ventricles (Fig. 6a). DeepHarmony successfully removed measurement bias and reduced variability, but not to the same extent as HACA3.

#### 3.1.5 Absolute volume difference percentage

**Fig. 7.**
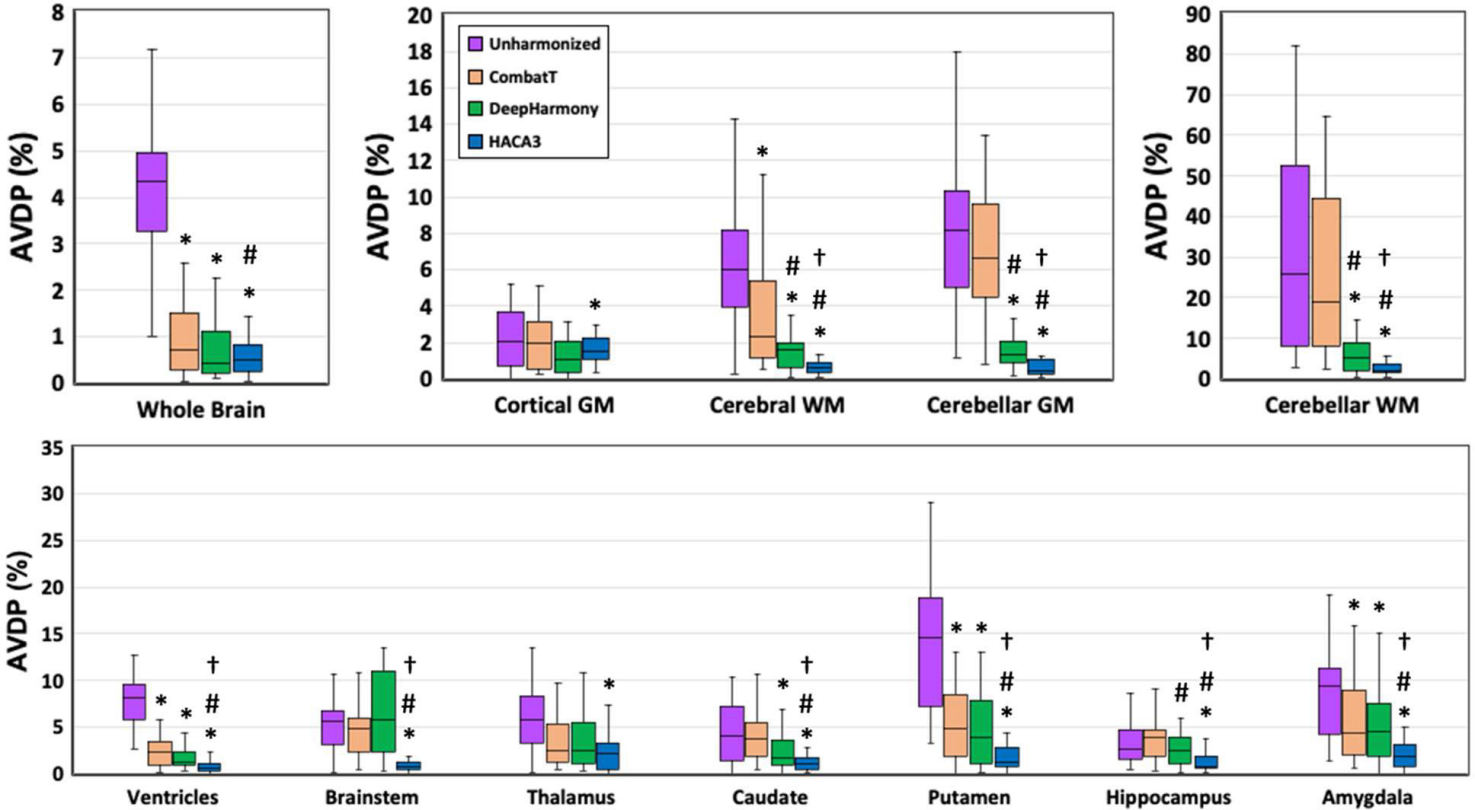
Absolute volume difference percentages (AVDP, %) of regional brain volumes between GRE and MPRAGE images of the testing cohort (N = 27). Paired t-tests of AVDP were conducted between the unharmonized images and the three other methods. *: *p_adj_* < 0.05 for significant improvement over unharmonized results. #: *p_adj_* < 0.05 for significant improvement over CombatT results. †: *p_adj_* < 0.05 for significant improvement over DeepHarmony results.

The AVDPs of regional brain volumes between GRE and MPRAGE images are reported in Figure 7 and Supplementary Table S2. HACA3 provided the smallest AVDP for most brain regions. For the ventricles, cerebral WM, cerebellar GM, cerebellar WM, brainstem, caudate, putamen, hippocampus, and amygdala, HACA3 yielded significantly lower AVDP than both the unharmonized results and the other harmonization techniques (*p_adj_* < 0.05). CombatT without EB and DeepHarmony also improved the AVDP from the unharmonized images in various regions, but to a lesser extent than HACA3. Particularly notable is the performance of HACA3 in ventricles, cerebellar GM, and cerebellar WM, where it substantially reduced the AVDP by more than 90%, a marked improvement over CombatT without EB and DeepHarmony.

To assess the impact of training set selection on AVDP, we performed an additional analysis using a different subset of 12 subjects for training from the cohort of 39. The remaining 27 subjects served as the testing dataset for both unharmonized data and data harmonized using CombatT without EB, DeepHarmony, and HACA3. The results were consistent with those from the original training and testing sets, with HACA3 producing the lowest AVDP across most brain regions, followed by DeepHarmony. These findings suggest that the study’s main conclusions remained robust to training and test sample selection. Note that because HACA3 is unsupervised, its results were only dependent on the test sample.

#### 3.1.6 Intra-class correlation

**Fig. 8.**
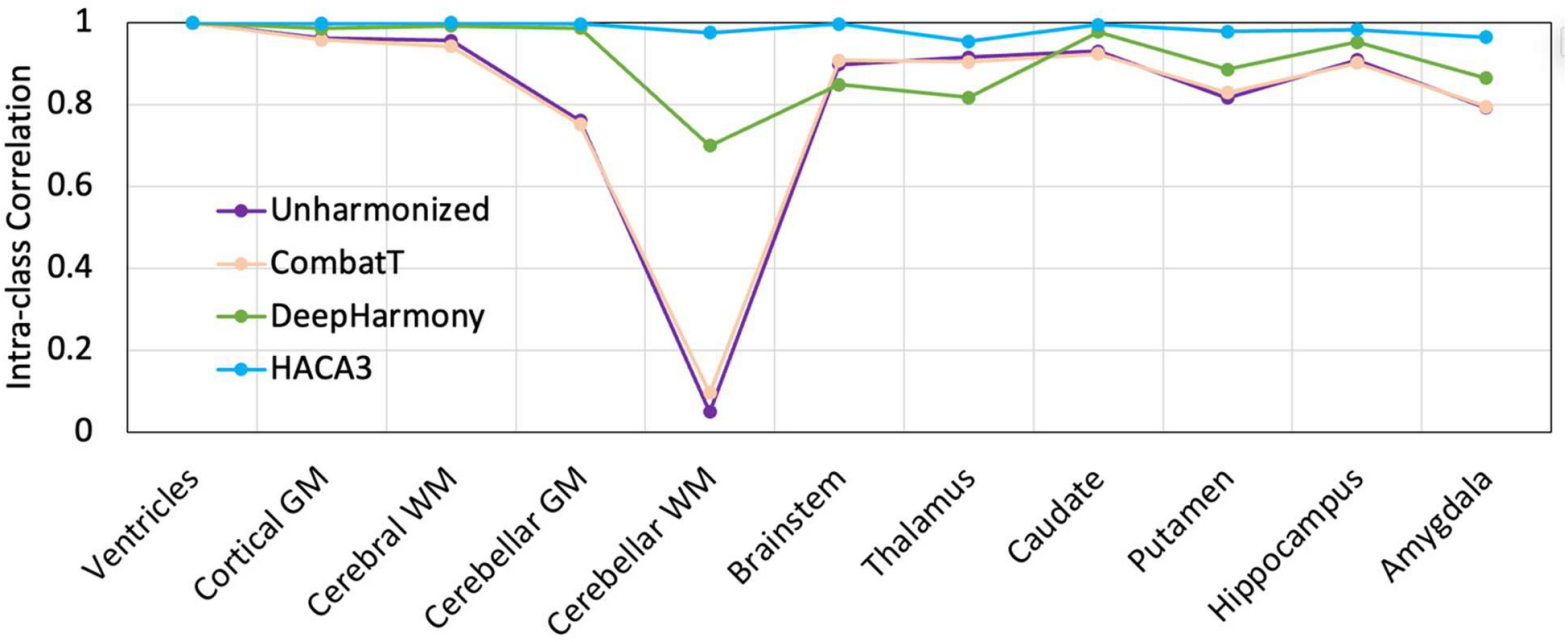
The intra-class correlation (ICC) between GRE and MPRAGE images without harmonization and with harmonization by CombatT, DeepHarmony, and HACA3 of the testing cohort (N = 27). Higher consistency represents better ICC between the GRE and MPRAGE images.

The ICC between GRE and MPRAGE volume measurements is presented in Figure 8. Unharmonized measurements and CombatT without EB exhibited variable consistency across different brain regions (ICC average ± standard deviation, range for unharmonized: 0.82 ± 0.27, 0.0490–0.9982; CombatT: 0.82 ± 0.25, 0.0954–0.9982), with some areas, such as the cerebellar WM, showing particularly low ICC values (ICC = 0.049 and 0.095, correspondingly). DeepHarmony offered improved consistency for most regions (ICC average 0.91 ± 0.10, range 0.6988–0.9988) but still showed reduced performance across the cerebellar WM and thalamus (ICC = 0.699 and 0.816, correspondingly). HACA3 consistently yielded the highest ICC values across all brain regions (ICC average 0.99 ± 0.02, range 0.9537–0.9997), demonstrating the most robust agreement between GRE and MPRAGE images post-harmonization.

#### 3.1.7 Coefficient of variation

The CV of brain volumes from GRE and MPRAGE images is shown as Bland–Altman plots in Supplementary Figure S4, with the average of CVs versus the difference of CVs. In the unharmonized data (Supplementary Fig. S4a), there was substantial variability in the CV, as indicated by the wide spread of points and the considerable distance from zero on the y-axis (standard deviation = 5.39 for the difference of CV). This indicates that the distribution of measurements differs for GRE and MPRAGE images. Harmonization with CombatT without EB (Supplementary Fig. S4b) reduced the variability somewhat, but there was still a notable spread of data points, and the limits of agreement were wide (standard deviation = 2.25 for the difference of CV). DeepHarmony (Supplementary Fig. S4c) showed further improvement in agreement for some regions, but others, such as the hippocampus, were still exhibiting a significant bias (standard deviation = 0.97 for the difference of CV). HACA3 (Supplementary Fig. S4d) demonstrated the smallest mean difference and the narrowest 95% confidence interval across all brain regions, indicating the highest level of agreement between CV of GRE and MPRAGE images after harmonization (standard deviation = 0.59 for the difference of CV). Note that the y-axis scale is reduced in all harmonized results compared with unharmonized data.

#### 3.1.8 Segmentation by SynthSeg

Brain regional volumes obtained from unharmonized GRE and MPRAGE images segmented by FreeSurfer and SynthSeg are reported in Table 4. Significant volume differences for FreeSurfer were observed in the ventricles, cerebral WM, cerebellar GM, cerebellar WM, thalamus, caudate, putamen, and amygdala. In contrast, SynthSeg revealed significant differences for the ventricles, cortical GM, cerebral WM, cerebellar WM, brainstem, thalamus, putamen, and amygdala. These results demonstrate that despite the fact that SynthSeg employs a domain randomization approach for segmentation training to improve model generalizability and robustness to contrast variation, it does not eliminate the need for harmonization.

**Table 4.** Volumes (cm^3^, mean ± standard deviation) of brain regions for GRE and MPRAGE images and their corresponding absolute volume difference percentages (AVDP, %, mean ± standard deviation) of the testing cohort (N = 27), **segmented by FreeSurfer and SynthSeg** for unharmonized images.

	Segmented by FreeSurfer			Segmented by SynthSeg		
Brain regions	GRE	MPRAGE	AVDP	GRE	MPRAGE	AVDP
Whole brain	1213 ± 137	1163 ± 127*	4.22 ± 1.31	1218 ± 128	1209 ± 128*	0.75 ± 0.40^#^
Ventricles	17.0 ± 7.0	18.4 ± 7.2*	8.66 ± 3.63	19.3 ± 7.2	20.0 ± 7.3*	3.98 ± 0.81^#^
Cortical GM	466 ± 43	462 ± 45	2.22 ± 1.59	524 ± 50	519 ± 49*	1.02 ± 0.83^#^
Cerebral WM	513 ± 80	480 ± 66*	6.49 ± 3.52	461 ± 58	458 ± 60*	0.91 ± 0.68^#^
Cerebellar GM	110 ± 14	107 ± 12*	7.94 ± 4.27	112 ± 11	113 ± 11	1.23 ± 0.86^#^
Cerebellar WM	40.1 ± 11.4	28.5 ± 3.1*	31.7 ± 24.7	32.3 ± 3.6	30.5 ± 3.3*	6.01 ± 2.52^#^
Brainstem	22.1 ± 2.7	22.3 ± 3.2	5.12 ± 2.71	21.7 ± 2.5	21.5 ± 2.6*	1.64 ± 0.88^#^
Thalamus	17.1 ± 2.1	16.4 ± 2.1*	5.72 ± 3.56	15.6 ± 1.6	15.2 ± 1.6*	2.78 ± 1.86^#^
Caudate	6.40 ± 0.81	6.68 ± 0.84*	4.79 ± 4.08	7.96 ± 0.89	8.00 ± 0.86	1.17 ± 1.05^#^
Putamen	8.56 ± 1.18	9.80 ± 1.52*	14.3 ± 6.91	11.1 ± 1.3	11.3 ± 1.2*	2.45 ± 2.14^#^
Hippocampus	8.70 ± 0.94	8.76 ± 0.90	3.55 ± 2.87	9.13 ± 0.79	9.14 ± 0.80	1.15 ± 1.25^#^
Amygdala	3.26 ± 0.51	3.42 ± 0.46*	9.08 ± 5.37	3.63 ± 0.42	3.75 ± 0.44*	3.49 ± 2.51^#^

* : *p_adj_* < 0.05, for paired t-tests of volumes conducted between the GRE and MPRAGE images. The volumes for the whole brain are presented and compared between GRE and MPRAGE images (*: *p-value* < 0.05). #: *p_adj_* < 0.05 for paired t-tests of AVDP conducted between FreeSurfer and SynthSeg approaches. The AVDPs for the whole brain are presented and compared between FreeSurfer and SynthSeg approaches (#: *p-value* < 0.05).

### 3.2 Sensitivity analysis

**Fig. 9.**
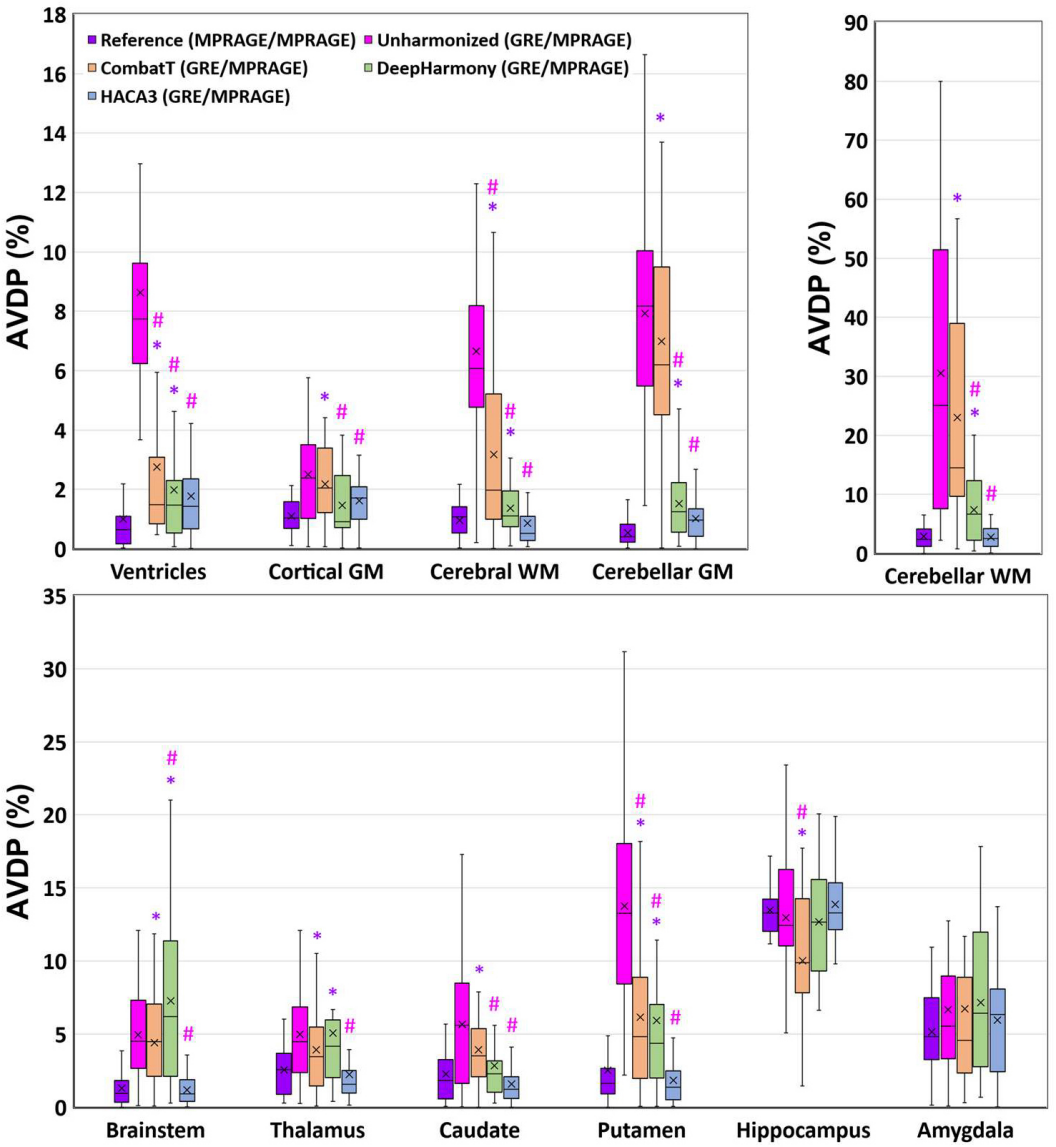
Boxplots of the absolute volume difference percentages (AVDP, %) of the testing cohort (N = 27) for the unharmonized images and harmonized data with simulated atrophy by CombatT, DeepHarmony, and HACA3. Paired t-tests of AVDP were conducted between the MPRAGE/MPRAGE of unharmonized data and the GRE/MPRAGE of harmonized data and between the GRE/MPRAGE of unharmonized data and the GRE/MPRAGE of harmonized data. ^*^: *p_adj_* < 0.05: significant difference between AVDP of the MPRAGE/MPRAGE of unharmonized data and the GRE/MPRAGE of harmonized data. #: *p_adj_* < 0.05: significant difference between the AVDP of the GRE/MPRAGE of unharmonized data and the GRE/MPRAGE of harmonized data. MPRAGE/MPRAGE: AVDP between MPRAGE without atrophy and MPRAGE with atrophy images. GRE/MPRAGE: AVDP between GRE without atrophy and MPRAGE with atrophy images.

This analysis examined the ability to detect volume changes in heterogeneous longitudinal data with simulated atrophy. AVDPs for various brain regions were computed between GRE (without atrophy) and MPRAGE (with simulated atrophy) images (namely “GRE/MPRAGE”) to evaluate the differences in brain volumes under unharmonized conditions and after harmonization using CombatT without EB, DeepHarmony, and HACA3. We compared the AVDPs from each of the harmonization methods (“GRE/MPRAGE”) with the AVDPs from the unharmonized data (“GRE/MPRAGE”) (Fig. 9; Supplementary Table S3). HACA3 consistently shows the smallest AVDP across most brain regions, especially in regions such as cerebral WM (0.87 ± 0.85), cerebellar GM (1.01 ± 0.82), and the brainstem (1.19 ± 1.04). DeepHarmony also demonstrates significant improvements in harmonization, achieving lower AVDPs compared with unharmonized data, with notable performance in cortical GM (1.46 ± 1.11) and cerebral WM (1.37 ± 0.89). NeuroCombat, while effective, exhibits higher variability in some regions, such as cerebellar WM (23.07 ± 17.5), compared with the other methods.

In addition, the “MPRAGE/MPRAGE” scenario represents the reference condition where the only variation between the images is the simulated atrophy within the same contrast (Fig. 9; Supplementary Table S3). Non-significant differences in AVDPs relative to the reference indicate good harmonization performance. The results showed that the AVDPs had no significant differences between HACA3 and the reference comparisons. DeepHarmony exhibited non-significant differences in three brain regions (Cortical GM, Caudate, and Amygdala) compared with the unharmonized data, while neuroCombat showed non-significant differences only in the Amygdala when compared with the unharmonized data.

In terms of overall sensitivity for detecting hippocampal atrophy, HACA3 demonstrated the largest effect size. Specifically, the effect sizes (Cohen’s d) (J. Cohen, 1988) for HACA3, DeepHarmony, and CombatT were 5.69, 3.33, and 1.90, respectively, when comparing hippocampal volumes between GRE scans without hippocampal atrophy and MPRAGE scans with hippocampal atrophy. For reference, the effect size for hippocampal atrophy using images with the same contrast (MPRAGE) without harmonization was 6.39, while the effect size for contrasting image types (GRE and MPRAGE) without harmonization was 2.54. While CombatT reduced the effect size of hippocampal atrophy, it also mitigated the risk of falsely detecting atrophy in unaffected structures (e.g., ventricles, cerebral WM, and putamen), a notable issue observed in the unharmonized results (Fig. 9).

We further compared the scenarios for CombatT and CombatU without EB, using GRE images (without hippocampal atrophy) and MPRAGE images (with hippocampal atrophy) (Supplementary Table S4). With CombatT, differences in hippocampal volumes were correctly detected between the GRE and MPRAGE images. Conversely, without trained models, the average volumes derived from the GRE images (without atrophy) were found to be equivalent to those from corresponding brain regions in the MPRAGE images (with atrophy), leading to non-significant p-values (*p_adj_* = 1.00) across all brain regions. This observation suggests a potential limitation of neuroCombat without trained models in differentiating between images with and without brain volume changes when there is a systematic bias in the imaging data, particularly in the absence of training models for non-atrophic data.

### 3.3 Combination of harmonization methods

Regional brain volumes and AVDP values as measured from GRE and MPRAGE images, analyzed by the DeepHarmony and HACA3 harmonization methods in combination with CombatT without EB, are presented in Supplementary Figure S5 and Supplementary Table S5. Paired t-tests of AVDP were conducted between DeepHarmony versus combined DeepHarmony and CombatT approaches and between HACA3 versus combined HACA3 and CombatT approaches. The findings indicate that combining DeepHarmony with CombatT did not result in a statistically significant improvement in harmonization effectiveness, as the AVDPs for the combined approach did not show significant differences when compared with DeepHarmony alone across all brain regions. In contrast, for the HACA3 harmonization method combined with CombatT, a significant improvement in AVDP was observed specifically for cortical GM (from 1.61 ± 0.74 to 0.57 ± 0.39). However, for the other brain regions, the combination of HACA3 and CombatT did not significantly alter the AVDP compared with HACA3 alone, indicating that the added benefit of combining these methods may be region specific. The ICC for DeepHarmony and HACA3 when compared with and without CombatT was 0.999 and 0.975, respectively, further indicating that the combination demonstrated limited improvement in measurement consistency (Supplementary Fig. S6). However, there was a reduction in the bias within VDP measurements when using HACA3 with CombatT. Conversely, there was a slight increase in bias when using DeepHarmony with CombatT.

## 4 Discussion

### 4.1 Comparison of harmonization methods

Reducing nuisance variation using data harmonization is important when working with heterogeneous imaging data, essential for maintaining consistency and reliability. Consistent volumetric measurement is a cornerstone in neuroimaging analysis, particularly when comparing data across different MRI scanners or protocols (Garcia-Dias et al., 2020; Pomponio et al., 2020; Shinohara et al., 2017; Smith et al., 2019). Across the three methods, HACA3 using MPRAGE as a target contrast demonstrated the best overall performance for MRI volumetric analyses. It substantially reduced AVDPs and VDPs with respect to both the values and the amount of variation across various brain regions and scans. High ICC values were also observed across all tested brain regions. The improvement in measurement consistency across various regions demonstrates the ability to improve the reliability of volumetric analyses in studies involving MRI data from multiple scans or scanners (Saponaro et al., 2022; Tian et al., 2022; Y.-W. Wang et al., 2023; H. Xu et al., 2023). Previous studies have also shown that HACA3 outperforms other contemporary image harmonization methods with respect to image quality, particularly in managing varied contrast and structural details in MR images (Zhang et al., 2023a, 2023b). A noted shortcoming of HACA3’s performance was that a small bias in cortical GM volume measurement remained after harmonization (Fig. 6), which is an important region of interest in many neuroimaging studies. Additional studies are needed to determine whether these results are replicated in other datasets. This bias was removed when combining HACA3 with neuroCombat.

NeuroCombat performed well at removing biases in measurements between the two MRI contrasts, but measurement variability was high. One significant benefit of neuroCombat is its ability to conditionally incorporate covariates such as age and disease status, resulting in more consistent and comparable outcomes across different imaging sites and protocols (Clark et al., 2023; Marzi et al., 2024; Stamoulou et al., 2021; R. Wang et al., 2022). In addition, neuroCombat does not require large training datasets, making it more accessible and suitable for studies with limited sample sizes (Fortin et al., 2017, 2018; Johnson et al., 2006).

To compare supervised and unsupervised image harmonization methods, our experimental design intentionally reflects a realistic and practical scenario: “What if a user directly applies a published harmonization method to their own data?” Supervised methods require retraining using traveling subjects between these new sites, which necessitated the use of training images collected in an identical manner as our testing data. However, HACA3 was evaluated using pretrained weights that did not include images from our “evaluation sites.” Compared with DeepHarmony or CombatT, HACA3 can, therefore, be considered to be more applicable to real-world scenarios where collection of paired training data is not possible.

The difference in training dataset sizes between the unsupervised and supervised deep learning approaches was primarily determined by the inherent data requirements and learning paradigms of these methods, rather than an intentional imbalance in experimental design. Supervised approaches such as DeepHarmony rely strictly on paired traveling-subject scans, substantially limiting available training data. Empirical findings from the original DeepHarmony study (Dewey et al., 2019) indicate that a training set comprising eight subjects is sufficient for effective model performance. This is because DeepHarmony is optimized for 2D images and performs effectively with computationally efficient 2D networks, enabling a larger training dataset since each image slice is unique. To further enhance robustness against artifacts and variations, DeepHarmony employs three separately trained networks across axial, sagittal, and coronal orientations to predict final volumes. In our experiments, we increased this number to 12, which is larger than the training set reported in the DeepHarmony paper. This number also reflects an attainable goal for the requirement of paired data, which would require traveling subjects. In contrast, unsupervised methods such as HACA3 can leverage larger and diverse datasets without such pairing requirements. While we acknowledge that differences in dataset size may impact performance comparisons, they also reflect fundamental distinctions in how these models operate—unsupervised approaches inherently benefit from larger datasets due to their more flexible learning framework.

HACA3 provides greater flexibility than DeepHarmony because it can be trained using either single-contrast or multi-contrast data and does not require paired datasets. Although it is technically feasible to train HACA3 on paired data, the current implementation does not support configurations that involve only paired data or a mixture of paired and unpaired data. Modifying the method to accommodate such configurations would require a fundamental redesign of the algorithm, resulting in a version that differs from the one originally proposed and evaluated in this study. Using HACA3 exclusively with paired data is also inconsistent with its design assumptions, which are based on learning from unpaired or weakly aligned multi-site datasets. In addition, effective training of HACA3 generally requires a larger and more diverse dataset. A limited paired dataset, such as the one used for DeepHarmony, is unlikely to meet this requirement and may lead to suboptimal performance or even failure to converge during training. Future research that evaluates both methods under identical training conditions, with a specific focus on HACA3’s performance in more constrained training scenarios, would allow for a more balanced comparison and yields a deeper understanding of its robustness and generalizability.

The overall performance of DeepHarmony was in between that of neuroCombat and HACA3. Measurement bias and variability were reduced in consistency testing, although the variability was not reduced to the same extent as HACA3, and it demonstrated sensitivity to hippocampal atrophy in sensitivity testing. The primary disadvantage is that its training requires the availability of paired data (i.e., subjects scanned with both contrasts being used within the study). Collection of such data is not always possible or may be resource prohibitive. One might expect the performance of DeepHarmony to surpass that of HACA3, given that it is supervised. However, it should be noted that the size of the training dataset used in DeepHarmony was relatively small, using only 12 subjects. However, HACA3 used scans from 210 subjects from a variety of MRI scanners and acquisition protocols. It is possible that DeepHarmony’s performance would surpass that of HACA3 given additional training data. Because HACA3 does not require paired training data, the potential for leveraging large datasets from public sources is an important advantage.

The computational time substantially differed between each harmonization method utilized in this study. NeuroCombat was executed in R version 4.2.2 on a system with an AMD EPYC 7543 32-Core Processor and 2 GB of memory. The entire process, including model training and feature prediction on the test dataset, took approximately 1 minute. For DeepHarmony, training time for each model corresponding to different anatomical orientations (axial, sagittal, and coronal) was approximately 10 hours per direction on an NVIDIA K80 GPU, with 80 GB of memory allocated for GPU-accelerated processing. The inference time for each 3D image volume was about 1 minute per anatomical orientation, 3 minutes total. For HACA3, the training duration was strongly influenced by the size of the training dataset. In the current experiment, training across 21 sites spanned roughly 3 days on an NVIDIA A6000 GPU, utilizing 7 GB of memory. Once the model was fully trained, harmonizing a complete 3D volume required approximately 3 minutes.

### 4.2 Reference images for deep learning harmonization methods

The selection of the reference sequence in DeepHarmony and HACA3 influenced the measured volumes of brain structures. The extent of this impact may vary depending on the image segmentation tool employed. FreeSurfer is generally designed for T1-weighted images (Droby et al., 2021; Greer, 2013; Renvall et al., 2016; Ryu et al., 2019), but is optimized for MPRAGE due to its superior GM contrast to facilitate FreeSurfer’s cortical reconstruction algorithms (Akudjedu et al., 2018; Fujimoto et al., 2014; Knussmann et al., 2022). The MPRAGE sequence generally provides a better contrast-to-noise ratio and reduced artifacts, resulting in more accurate segmentation results with FreeSurfer (Fu et al., 2022; Goncalves Filho et al., 2023). While GRE images can also be processed with FreeSurfer, they are more susceptible to inhomogeneities and unwanted distortions such as susceptibility effects, depending on scanner settings and sequence parameters (Lutti et al., 2014; Reuter et al., 2010; van der Kouwe et al., 2008). Therefore, MPRAGE is often the preferred choice for FreeSurfer due to its compatibility with the FreeSurfer’s processing algorithms, although the final choice between GRE and MPRAGE may depend on the specific requirements of the research study and the available MRI equipment. Nevertheless, even with GRE as a target contrast, both DeepHarmony and HACA3 provided improved consistency of FreeSurfer volume measurement compared with unharmonized results. Future studies should explore alternative segmentation tools, such as FastSurfer (Henschel et al., 2020), ANTs (Avants et al., 2011), DeepBrain (Ranjbar et al., 2020), or SLANT (Huo et al., 2019) to assess the consistency of the observed effects across different segmentation frameworks.

### 4.3 Model selection in neuroCombat

The flexibility of neuroCombat is one of its key strengths, enabling the harmonization of diverse neuroimaging data types, including structural MRI, functional MRI, and diffusion-weighted imaging (Fortin et al., 2017, 2018; Orlhac et al., 2022; Richter et al., 2022). However, this flexibility is not without limitations. When employing the EB option, neuroCombat relies on the assumption that site-specific effects conform to certain prior distributions (specifically, a normal distribution for γ and an inverse gamma distribution for $\delta^{2}$), which may not be universally applicable (Fortin et al., 2017, 2018). In our dataset, this assumption led to suboptimal performance when EB was applied, as compared with results where it was not used. Specifically, neuroCombat with EB assumes that batch effects follow a shared prior distribution across subjects and applies shrinkage estimation to stabilize variance, which is particularly beneficial when sample sizes are small or batch effects are highly variable. However, in our case, the batch effects between GRE and MPRAGE images appear to be relatively systematic and consistent, meaning that EB shrinkage may have led to overcorrection or misattribution of variance that should have been retained. By disabling EB, neuroCombat instead applies a direct batch correction without imposing prior assumptions on the distribution of batch effects, which led to better harmonization in this specific context.

While neuroCombat is a highly valuable tool for the harmonization of neuroimaging data across various sites or timeframes, incorrect application can yield suboptimal results. This was evident when it was used without training data in the sensitivity analysis, where the hippocampal atrophy was completely removed during the harmonization step (Supplementary Table S4). Although it is possible to use neuroCombat without a training dataset, this approach has specific pros and cons. Without a training dataset, the process is simpler because there is no need to collect and preprocess additional data. It can be applied directly to the available data without delay (Bell et al., 2022; Richter et al., 2022). However, within our dataset, the absence of a dedicated training dataset limited neuroCombat’s ability to robustly model and correct for batch effects, resulting in suboptimal harmonization outcomes. Caution in applying neuroCombat without training is warranted, particularly when systematic batch effects may be associated with biological effects.

In our analysis, neuroCombat without EB and using a training dataset yielded the most favorable results among the neuroCombat models tested. These models assume that the two contrasts (GRE and MPRAGE) originate from independent cohorts scanned at distinct time points across two sites, consistent with our approach in the HACA3 method, where these data were treated as distinct groups rather than longitudinal observations. While this approach diverges from neuroCombat’s original design for cross-sectional harmonization, Longitudinal ComBat, which accounts for within-subject correlations over time, may be more appropriate for datasets with true temporal changes (Beer et al., 2020). However, Longitudinal ComBat requires at least two scans per scanner to accurately estimate scanner effects, and it is crucial that sample sizes and covariates are sufficiently balanced across scanners to ensure unbiased estimation of these effects (Beer et al., 2020). Although not shown, our experiments with Longitudinal ComBat revealed higher AVDPs across most brain regions than the cross-sectional approach, possibly due to the complexity of preserving temporal trends. In cases where temporal changes are minimal, neuroCombat’s simpler approach, which treats each cohort as independent, may reduce the risk of overfitting and result in more reliable harmonization (Richter et al., 2022). Furthermore, in datasets characterized by strong batch effects, neuroCombat’s more robust correction of site-related differences may outperform Longitudinal ComBat, which prioritizes maintaining within-subject variation (Richter et al., 2022). We, therefore, reported only the cross-sectional neuroCombat results here but recognize the need for further investigations into optimizing this approach for longitudinal data.

Because the data used in this study involved paired scans from the same subjects, covariates were not included in the primary analyses using CombatT. We verified that the incorporation of biological covariates into the model did not improve harmonization by using the neuroHarmonize software package (Pomponio et al., 2019), as implemented by Pomponio et al. (2020), which employs Generalized Additive Models (GAMs) to model covariates (age, sex, and body mass index [BMI]) with either linear or nonlinear effects (Pomponio et al., 2020). The incorporation of covariates was evaluated on the two datasets used for consistency analysis and atrophy sensitivity. Our findings in this secondary analysis suggested that adjusting for either linear or nonlinear covariate effects did not significantly improve neuroCombat’s ability to harmonize volumetric measurements. Although the design of this study provided perfectly matched samples, obviating the need for modeling of covariates, studies where subject populations are different across sites might benefit from incorporating covariate adjustment to control for demographic or biological variability that could confound harmonization outcomes.

### 4.4 Atrophy simulation

Although measurement consistency across heterogeneous MRI acquisitions is important, this consistency should not come at the expense of sensitivity to biological variations and changes. In testing the algorithms with simulated hippocampal atrophy, all three harmonization methods reduced AVDP across GRE and MPRAGE scans in most regions away from the hippocampus while retaining sensitivity to simulated changes in the hippocampus and adjacent structures such as the amygdala. The results of HACA3, however, most resembled the volume measurements from homogeneous MPRAGE scans with and without atrophy. NeuroCombat showed volume changes greater than 6% in the cerebellar GM, cerebellar WM, putamen, and amygdala, while DeepHarmony detected volume changes greater than 6% in cerebellar WM, brainstem, and amygdala. These regions were also found to have relatively large changes in the consistency analysis. Such false positives might potentially obscure the identification of structures that actually underwent volumetric change or alternatively might lead to inaccurate conclusions about the data. Nevertheless, these detected changes were still a substantial improvement over using only unharmonized data.

### 4.5 Limitations

This comparison study was performed on a relatively small dataset and portions were held out to be used as training and test data. It is possible that a large sample size would have benefited the performance of neuroCombat and/or DeepHarmony. Another limitation was that the effect of geometric distortions was not considered. Although distortions are more commonly associated with echoplanar imaging acquisitions used in diffusion and functional MRI, geometric distortions also occur in structural imaging and have been shown to potentially affect volumetric analyses (Lee et al., 2019; Thaler et al., 2023). The scans employed in this study used the scanner product corrections performed during acquisition. The image-based harmonization algorithms used in this study do not currently address differences in geometric distortion between acquisitions. NeuroCombat could potentially address differences if the distortions manifest as systematic biases in the volume measurements of the various anatomical regions. A previous study found that distortion correction using a geometric phantom scan improved measurement precision in a multicenter study (Nanayakkara et al., 2022). Additional investigations into the combination of geometric distortions and harmonization algorithms are needed to better understand its impact. Finally, the GRE and MPRAGE images used in this study represent a relatively strong contrast change that might represent a more challenging harmonization problem than what might be encountered in some heterogeneous imaging studies. Although this allowed the differences between the harmonization approaches to be accentuated, the differences may be more subtle depending on the datasets.

A limitation of this study is that it focuses on harmonizing differences in acquisition settings within the same scanning session, rather than addressing the broader range of site effects that may be encountered in multi-center studies. While this controlled setting allows for a systematic evaluation of DeepHarmony, HACA3, and neuroCombat within a well-defined environment, real-world multi-center studies may introduce additional sources of variability, including differences in scanner manufacturers, field strength, temporal shifts, and recruitment protocols. Our primary objective was to assess the effectiveness of published approaches in harmonizing systematic contrast differences, which pose a significant challenge in retrospective neuroimaging analyses. Generalizing our findings to broader multi-site studies requires further investigation. In addition, while our results highlight the potential of unsupervised deep learning for harmonization, further research is needed to evaluate its robustness across diverse imaging sites, scanner hardware, and acquisition protocols, enabling an improved assessment of the scalability and adaptability of these harmonization methods.

While deep learning-based harmonization methods such as HACA3 offer promising performance in reducing site-related variability in brain medical imaging, several important limitations must be considered when applying pretrained models to new datasets. First, there is a potential risk of domain shift, where the characteristics of the new data (e.g., scanner type, image contrast, or subject pathology) differ from those seen during model training, potentially degrading harmonization performance. Although HACA3 performed reasonably well on the independent dataset used in this evaluation, it may not generalize well across all datasets with unseen imaging protocols or demographic and clinical variability. In particular, the generalizability of these models across different clinical populations remains a challenge. For instance, HACA3 would be unlikely to perform well on data collected from subjects with brain tumors or traumatic brain injury, as these conditions involve substantially different pathological presentations. To mitigate these issues, users are advised to evaluate the compatibility between their data and the pretrained model’s training domain, conduct preliminary validation, and, when appropriate, fine tune the model using a representative subset of their dataset to improve domain adaptation and enhance overall robustness.

## 5 Conclusion

This study compares image-based neural network methods and statistical methods for brain volume harmonization. Algorithms were evaluated based on their ability to reduce volume discrepancies between images acquired using different protocols, a common issue in multicenter MRI studies. Overall, HACA3 surpassed DeepHarmony and neuroCombat in achieving consistency, proving especially advantageous in situations where paired training data are unavailable. For volumetric studies involving heterogeneous MRI acquisitions, the use of any harmonization method was generally shown to be beneficial in improving measurement consistency when compared with results generated without harmonization and should, therefore, be an essential component of data analysis procedures. Future studies with disease-specific cohorts and clinical markers will be essential to assess whether harmonized data improve associations with phenotypes, disease progression, or diagnostic accuracy, which would be particularly valuable for clinical applications.

## Supplementary Material

Supplementary Material

## Data Availability

Code for the neuroCombat algorithm is available from https://github.com/Jfortin1/neuroCombat_Rpackage. Code for the DeepHarmony algorithm is available from https://gitlab.com/iacl/synmi. Code for the HACA3 algorithm is available from https://github.com/lianruizuo/haca3. Additional evaluation code is available upon request to Dr. Yuan-Chiao Lu and Dr. Dzung L. Pham. Data will be made available upon request to Dr. Daniel S. Reich.
