# Supplementary material for: An evaluation of image-based and statistical techniques for harmonizing brain volume measurements
Source: Imaging Neurosci (Camb). 2025 Jul 14;3:IMAG.a.73. doi: 10.1162/IMAG.a.73 (PMC12330838; doi:10.1162/IMAG.a.73)
Supplement: Supplementary Material [file IMAG.a.73_supp.pdf]

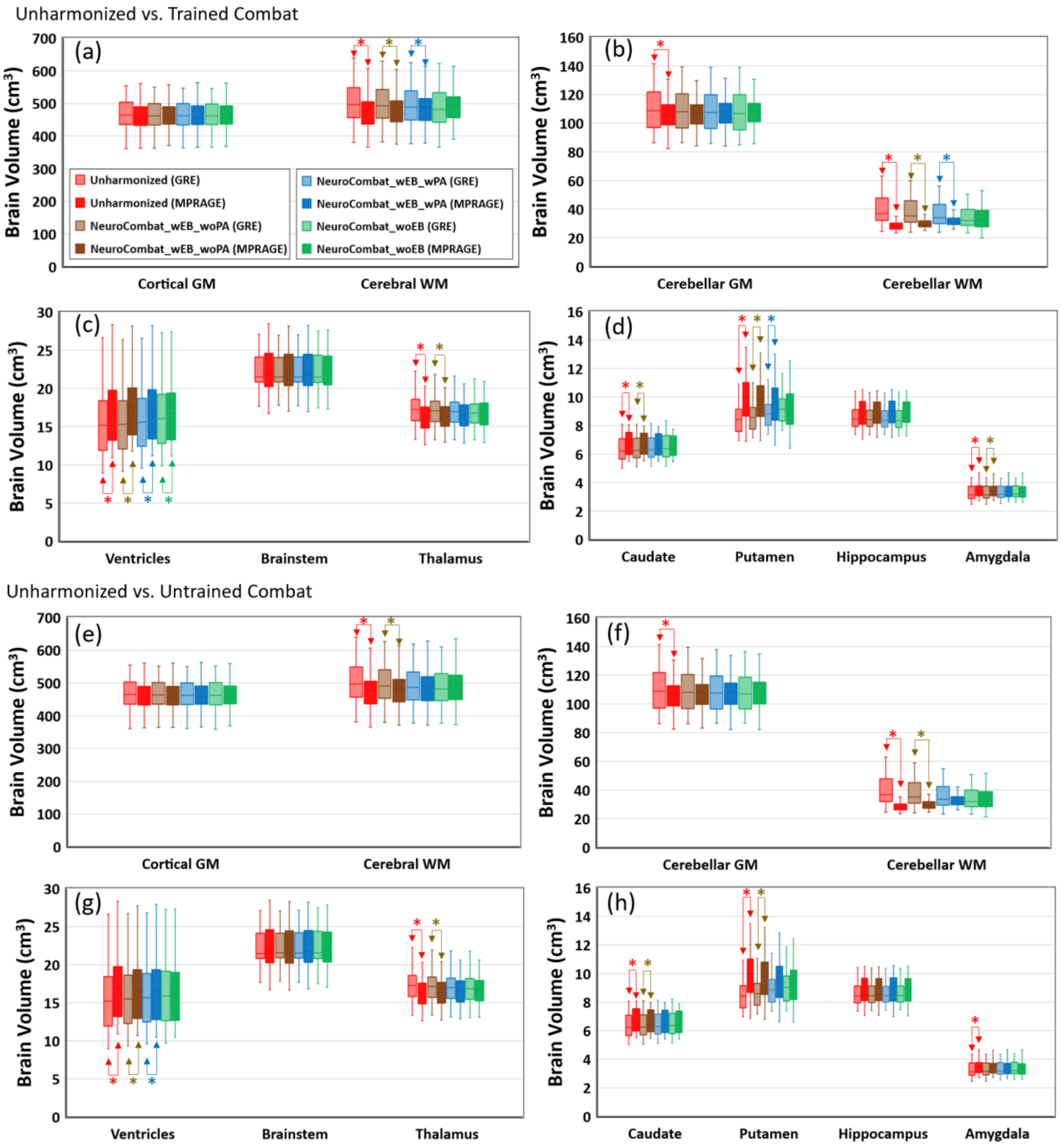

Supplementary Figure 1. Comparisons of brain regional volumes between GRE and MPRAGE of the testing cohort (N=27) for unharmonized data, neuroCombat with empirical Bayes and without parametric adjustments, neuroCombat with empirical Bayes and with parametric adjustments, and neuroCombat without empirical Bayes. (a)-(d) neuroCombat with a training set (CombatT). (e)-(h) neuroCombat without trained models (CombatU). *: $p_{adj} < 0.05$ based on the paired two-sample t-tests of brain volumes.

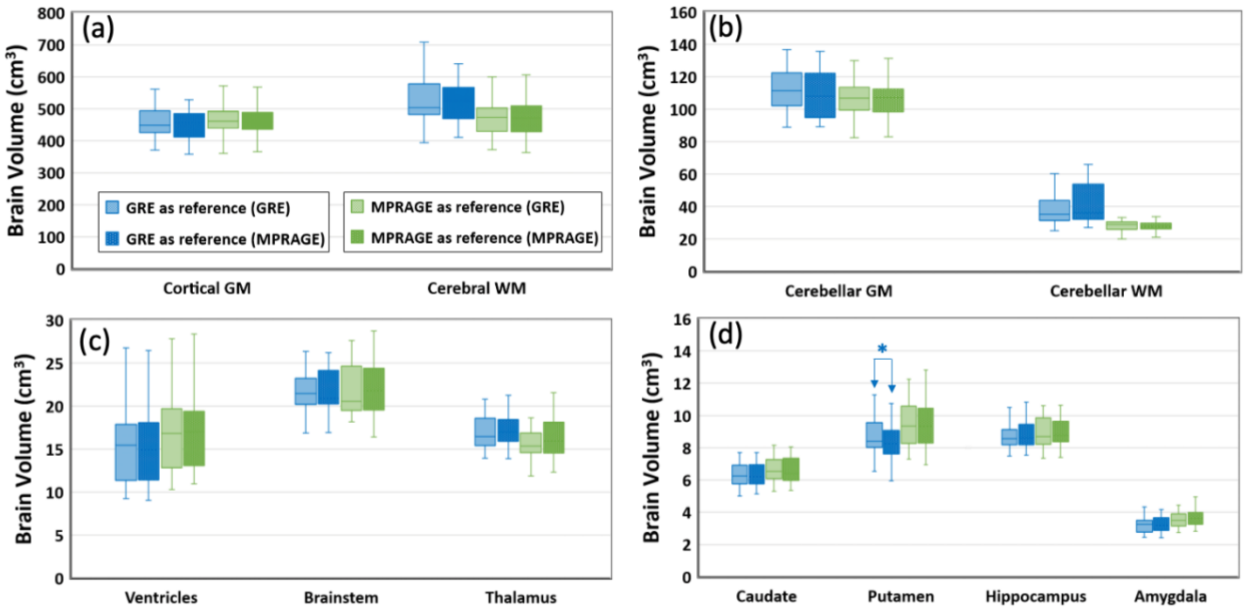

Supplementary Figure 2. Comparisons of brain regional volumes between GRE and MPRAGE of the testing cohort (N=27) determined from DeepHarmony harmonization results. *: p_{adj} < 0.05 based on the paired two-sample t-tests.

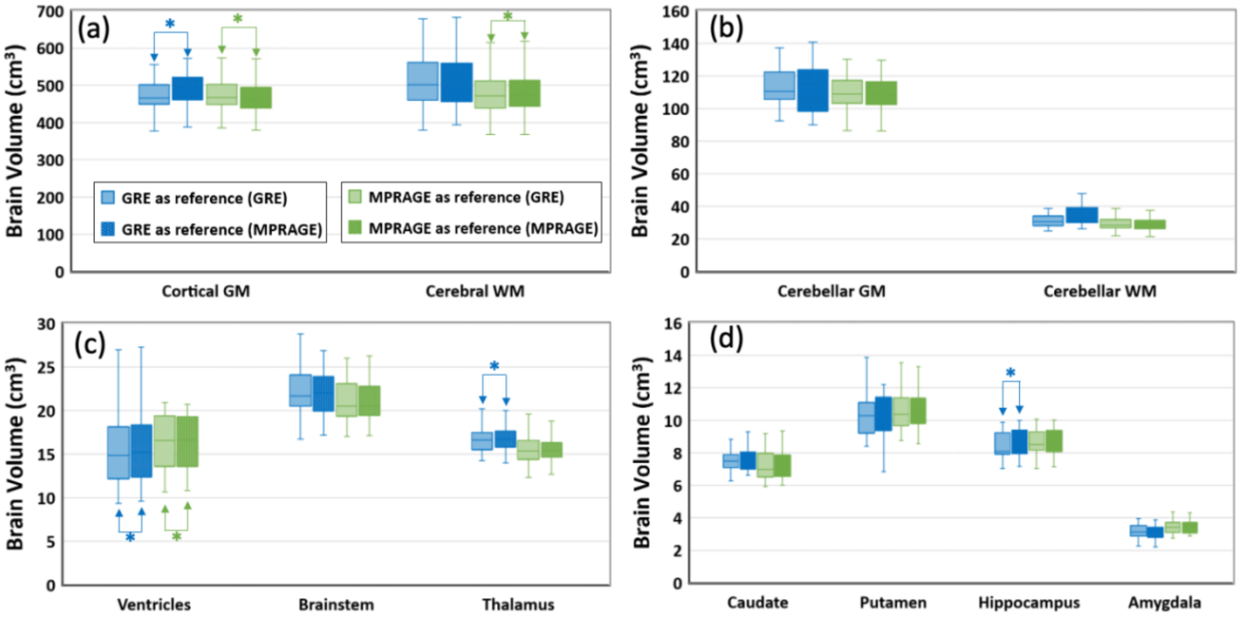

Supplementary Figure 3. Comparisons of brain regional volumes between GRE and MPRAGE of the testing cohort (N=27) determined from HACA3 harmonization results. *: $p_{adj} < 0.05$ based on the paired two-sample t-tests.

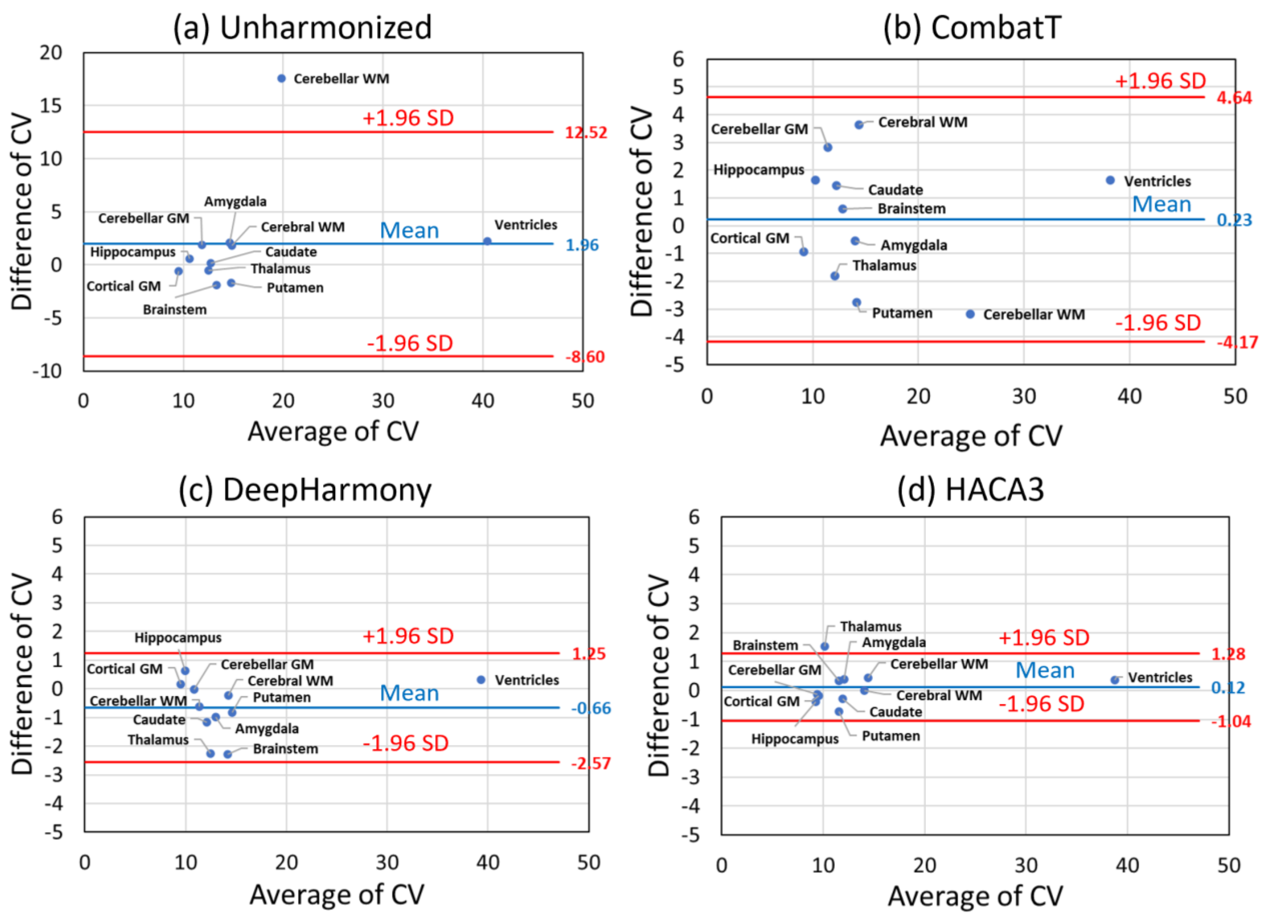

Supplementary Figure 4. Bland-Altman plots of the CV measurements by 11 brain regions of GRE and MPRAGE images without harmonization (a) and with harmonization by neuroCombat (b), DeepHarmony (c), and HACA3 (d) of the testing cohort (N=27). SD: Standard deviation.

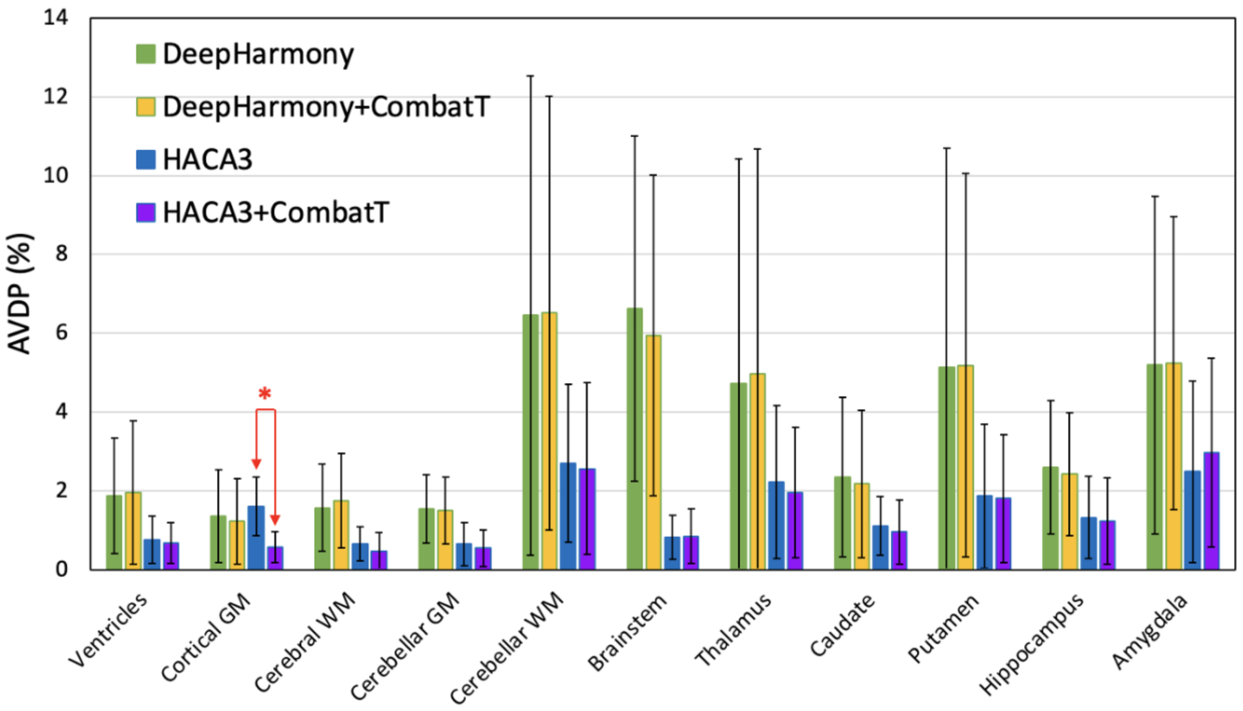

Supplementary Figure 5. Absolute volume difference percentages (AVDP, %) of the regional brain volumes between GRE and MPRAGE images of the testing cohort (N=27). *: $p_{adj} < 0.05$ for paired t-tests of AVDP conducted between the DeepHarmony+CombatT and DeepHarmony alone and between HACA3+CombatT and HACA3 alone.

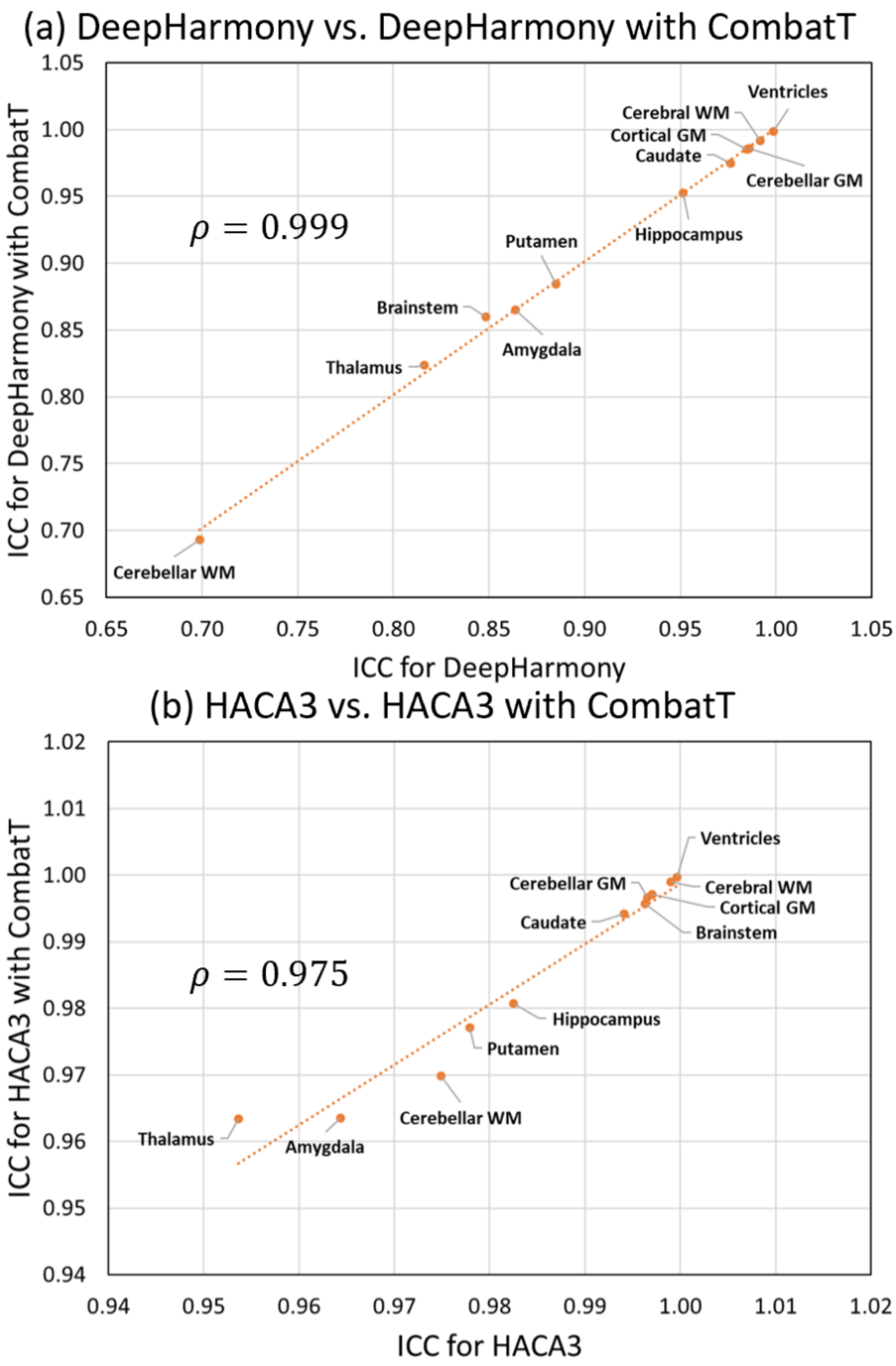

Supplementary Figure 6. Intra-class correlation (ICC) for combined DeepHarmony/HACA3 and CombatT approaches versus DeepHarmony/HACA3 approach alone, correspondingly, for the 11 brain regions of the testing cohort (N=27). (a) DeepHarmony with CombatT versus DeepHarmony alone. (b) HACA3 with CombatT versus HACA3 alone. ρ: Correlation coefficient between the ICC measurements.

Supplementary Table 1. Demographic characteristics of the subjects. Two-sample tests comparing age, height, weight, and BMI between the training and testing datasets showed no significant differences (*p-values* > 0.05) between the groups. A Chi-Square test revealed no significant difference (*p-value* = 0.62) in the distribution of male and female subjects between the training and testing datasets.

	Training Set (N=12)	Testing Set (N=27)	P-value
Age (years)	39.7±12.3	34.9±8.6	0.17
Sex (M/F)	5/7	9/18	0.62
Height (m)	1.72±0.10	1.72±0.09	0.94
Weight (kg)	80.8±24.6	74.2±11.4	0.25
BMI (kg/m^2)	27.3±7.8	25.1±3.3	0.21

Supplementary Table 2. Absolute volume difference percentages (AVDP, %, mean ± standard deviation) of regional brain volumes between GRE and MPRAGE images of the testing cohort (N=27). Paired t-tests of AVDP were conducted between the unharmonized images and the three other methods. *: $p_{adj} < 0.05$ for significant improvement over unharmonized results. #: $p_{adj} < 0.05$ for significant improvement over CombatT results. †: $p_{adj} < 0.05$ for significant improvement over DeepHarmony results. The averages of the AVDP values for the 11 brain regions, along with the AVDP values for the whole brain, were presented and compared to unharmonized data (*: *p-value* < 0.05), CombatT (#: *p-value* < 0.05), and DeepHarmony (†: *p-value* < 0.05). Bold: Lowest mean value of AVDP among the four scenarios for each brain region and the whole brain.

Brain regions	Unharmonized	CombatT	DeepHarmony	HACA3
Whole Brain	4.22±1.31	1.14±1.18*	0.73±0.67*	**0.58±0.39***#
Ventricles	8.66±3.63	2.74±2.75*	1.87±1.46*	**0.76±0.61***#†
Cortical GM	2.22±1.59	2.06±1.45	**1.35±1.18**	1.61±0.74*
Cerebral WM	6.49±3.52	3.35±2.91*	1.57±1.11*#	**0.66±0.43***#†
Cerebellar GM	7.94±4.27	7.06±4.27	1.54±0.87*#	**0.65±0.54***#†
Cerebellar WM	31.68±24.67	24.09±18.83	6.46±6.08*#	**2.70±2.00***#†
Brainstem	5.12±2.71	4.50±2.83	6.63±4.37	**0.82±0.56***#†
Thalamus	5.72±3.56	3.70±3.40	4.72±5.70	**2.22±1.94***
Caudate	4.79±4.08	3.98±2.50	2.35±2.02*	**1.11±0.74***#†
Putamen	14.34±6.91	6.58±6.19*	5.13±5.56*	**1.87±1.83***#†
Hippocampus	3.55±2.87	4.12±3.09	2.60±1.70#	**1.32±1.05***#†
Amygdala	9.08±5.37	6.31±5.81*	5.20±4.28*	**2.49±2.31***#†
Average	9.05±2.41	6.23±2.42*	3.58±1.59*#	**1.47±0.45***#†

Supplementary Table 3. Absolute volume difference percentages (AVDP, %, mean ± standard deviation) of regional brain volumes between MPRAGE (**without atrophy** of hippocampus) and MPRAGE (**with simulated atrophy** of hippocampus) images (Column name: MPRAGE/MPRAGE) and between GRE (**without atrophy** of hippocampus) and MPRAGE (**with simulated atrophy** of hippocampus) images (Column name: GRE/MPRAGE) of the testing cohort (N=27). Paired t-tests of AVDP were conducted between the MPRAGE/MPRAGE of unharmonized data and the GRE/MPRAGE of harmonized data and between the GRE/MPRAGE of unharmonized data and the GRE/MPRAGE of harmonized data. *: $p_{adj} < 0.05$: significant difference between AVDP of the MPRAGE/MPRAGE of unharmonized data and the GRE/MPRAGE of harmonized data. #: $p_{adj} < 0.05$: significant difference between the AVDP of the GRE/MPRAGE of unharmonized data and the GRE/MPRAGE of harmonized data. The averages of the AVDP values for the 11 brain regions, along with the AVDP values for the whole brain, were presented and compared to unharmonized MPRAGE/MPRAGE (*: *p-value* < 0.05) and GRE/MPRAGE (#: *p-value* < 0.05).

Brain regions	Unharmonized		CombatT	DeepHarmony	HACA3
	MPRAGE/MPRAGE	GRE/MPRAGE	GRE/MPRAGE	GRE/MPRAGE	GRE/MPRAGE
Whole Brain	0.66±0.42	4.63±1.34	1.18±1.10*#	0.65±0.52#	0.91±0.49#
Ventricles	1.01±1.48	8.63±4.70	2.75±3.80*#	1.98±1.80*#	1.77±1.29#
Cortical GM	1.10±0.55	2.50±1.71	2.18±1.32*	1.46±1.11#	1.62±0.92#
Cerebral WM	0.96±0.59	6.66±3.46	3.17±2.93*#	1.37±0.89*#	0.87±0.85#
Cerebellar GM	0.54±0.44	7.93±4.08	6.99±4.24*	1.52±1.06*#	1.01±0.82#
Cerebellar WM	2.89±2.22	30.6±23.6	23.0±17.5*	7.37±6.58*#	2.76±1.97#
Brainstem	1.31±1.23	4.97±3.07	4.43±2.98*	7.27±5.54*#	1.19±1.04#
Thalamus	2.55±2.09	4.99±3.94	3.93±3.10*	5.08±4.45*	2.26±2.31#
Caudate	2.26±1.81	5.69±4.44	3.94±2.80*	2.83±2.62#	1.58±1.40#
Putamen	2.54±3.18	13.8±6.99	6.18±5.59*#	5.92±7.08*#	1.86±1.75#
Hippocampus	13.5±1.81	13.0±4.41	10.0±4.10*#	12.7±3.47	13.9±2.51
Amygdala	5.17±3.07	6.68±5.58	6.75±6.61	7.17±4.94	5.95±3.57

Supplementary Table 4. Regional brain volumes (cm^3, mean ± standard deviation) for the GRE (without atrophy) and MPRAGE (with atrophy of hippocampus) images of the testing cohort (N=27) harmonized by neuroCombat **with** versus **without the trained models**. *: $p_{adj} < 0.05$ for paired t-tests of volumes conducted between the GRE (without atrophy) and MPRAGE (with atrophy of hippocampus) images. The volumes for the whole brain were presented and compared between GRE (without atrophy) and MPRAGE (with atrophy of hippocampus) images.

Brain regions	CombatT without EB			CombatU without EB		
	GRE	MPRAGE (atrophy)	p-value	GRE	MPRAGE (atrophy)	p-value
Whole Brain	1189±133	1185±125	0.34	1186±128	1186±135	1.00
Ventricles	17.9±6.9	18.2±6.7	<0.01*	17.7±7.0	17.7±7.0	1.00
Cortical GM	464±40	462±44	0.37	462±43	462±43	1.00
Cerebral WM	498±80	496±62	0.72	496±73	496±73	1.00
Cerebellar GM	109±14	108±11	0.87	108±13	108±13	1.00
Cerebellar WM	34.3±7.9	35.1±8.5	0.72	34.5±8.1	34.5±8.1	1.00
Brainstem	22.2±2.9	22.3±2.8	0.54	22.2±2.9	22.2±2.9	1.00
Thalamus	16.7±1.9	16.9±2.3	0.26	16.8±2.1	16.8±2.1	1.00
Caudate	6.59±0.85	6.58±0.75	0.90	6.57±0.81	6.57±0.81	1.00
Putamen	9.28±1.17	9.09±1.30	0.18	9.16±1.27	9.16±1.27	1.00
Hippocampus	8.63±0.95	7.81±0.74	<0.01*	8.18±0.86	8.18±0.86	1.00
Amygdala	3.34±0.45	3.18±0.44	<0.01*	3.25±0.46	3.25±0.46	1.00

Supplementary Table 5. Volumes (cm^3, mean ± standard deviation) of brain regions for GRE and MPRAGE images and their corresponding absolute volume difference percentages (AVDP, %, mean ± standard deviation) of the testing cohort (N=27). *: $p_{adj} < 0.05$, for paired t-tests of AVDP conducted between the DeepHarmony+CombatT and DeepHarmony alone (Table 2, "MPRAGE as reference") and between HACA3+CombatT and HACA3 alone (Table 3, "MPRAGE as reference"). The volumes for the whole brain were presented and compared between GRE and MPRAGE images (*: *p-value* < 0.05).

Brain regions	DeepHarmony+CombatT			HACA3+CombatT		
	Vol_{GRE}	Vol_{MPRAGE}	AVDP	Vol_{GRE}	Vol_{MPRAGE}	AVDP
Whole Brain	1165±121	1159±121	0.78±0.67	1179±124	1179±123	0.34±0.26*
Ventricles	18.5±6.9	18.7±6.8	1.95±1.82	18.8±6.8	18.9±6.8	0.68±0.51
Cortical GM	466±42	464±42	1.23±1.08	473±43	473±43	0.57±0.39*
Cerebral WM	480±64	475±65	1.75±1.20	482±64	481±64	0.48±0.47
Cerebellar GM	107±11	106±11	1.50±0.86	110±10	110±10	0.54±0.47
Cerebellar WM	28.0±2.8	28.2±3.3	6.52±5.51	29.1±4.2	29.2±3.8	2.56±2.18
Brainstem	21.8±2.8	22.1±3.1	5.94±4.07	21.1±2.4	21.0±2.3	0.85±0.69
Thalamus	15.5±1.7	16.2±2.0	4.97±5.71	15.6±1.6	15.7±1.5	1.97±1.65
Caudate	6.69±0.72	6.63±0.80	2.18±1.86	7.24±0.80	7.23±0.82	0.96±0.81
Putamen	9.45±1.35	9.39±1.26	5.18±4.86	10.63±1.12	10.70±1.22	1.81±1.63
Hippocampus	8.90±0.86	8.88±0.83	2.43±1.56	8.59±0.73	8.60±0.79	1.24±1.08
Amygdala	3.60±0.42	3.56±0.45	5.23±3.72	3.42±0.39	3.50±0.41	2.97±2.40
